# Assessing Bacterial Populations in the Lung by Replicate Analysis of Samples from the Upper and Lower Respiratory Tracts

**DOI:** 10.1371/journal.pone.0042786

**Published:** 2012-09-06

**Authors:** Emily S. Charlson, Kyle Bittinger, Jun Chen, Joshua M. Diamond, Hongzhe Li, Ronald G. Collman, Frederic D. Bushman

**Affiliations:** 1 Department of Microbiology, University of Pennsylvania School of Medicine, Philadelphia, Pennsylvania, United States of America; 2 Pulmonary, Allergy and Critical Care Division, Department of Medicine, University of Pennsylvania School of Medicine, Philadelphia, Pennsylvania, United States of America; 3 Infectious Disease Division, Department of Medicine, University of Pennsylvania School of Medicine, Philadelphia, Pennsylvania, United States of America; Department of Biostatistics and Epidemiology, Perelman School of Medicine, University of Pennsylvania, Philadelphia, Pennsylvania, United States of America; Baylor College of Medicine, United States of America

## Abstract

Microbes of the human respiratory tract are important in health and disease, but accurate sampling of the lung presents challenges. Lung microbes are commonly sampled by bronchoscopy, but to acquire samples the bronchoscope must pass through the upper respiratory tract, which is rich in microbes. Here we present methods to identify authentic lung microbiota in bronchoalveolar lavage (BAL) fluid that contains substantial oropharyngeal admixture. We studied clinical BAL samples from six selected subjects with potential heavy lung colonization. A single sample of BAL fluid was obtained from each subject along with contemporaneous oral wash (OW) to sample the oropharynx, and then DNA was extracted from three separate aliquots of each. Bacterial 16S rDNA sequences were amplified and products analyzed by 454 pyrosequencing. By comparing replicates, we were able to specify the depth of sequencing needed to reach a 95% chance of identifying a bacterial lineage of a given proportion—for example, at a depth of 5,000 tags, OTUs of proportion 0.3% or greater would be called with 95% confidence. We next constructed a single-sided outlier test that allowed lung-enriched organisms to be quantified against a background of oropharyngeal admixture, and assessed improvements available with replicate sequence analysis. This allowed identification of lineages enriched in lung in some BAL specimens. Finally, using samples from healthy volunteers collected at multiple sites in the upper respiratory tract, we show that OW provides a reasonable but not perfect surrogate for bacteria carried into to the lung by a bronchoscope. These methods allow identification of microbes that can replicate in the lung despite the background due to oropharyngeal microbes derived from aspiration and bronchoscopic carry-over.

## Introduction

The identification of lung microbial inhabitants using sequence surveys presents challenges not found in surveys of most other body sites. Sampling is commonly carried out by bronchoscopy, but insertion of the bronchoscope into the lung requires passing the bronchoscope through the oropharynx, which has a high density of bacteria. Classical studies [1], [2], [3], [4] and a more recent sequence based study [5] establish that oropharyngeal carry-over can predominate in bronchoscopic samples. Despite these limitations, it remains highly attractive to bring the new deep sequencing methods to bear on understanding lung colonization and infection [6].

We previously reported an analysis of airway bacterial populations from six healthy individuals using a two bronchoscope sampling procedure [5], in which the first bronchoscope was inserted only to the glottis to sample the microbiota encountered prior to entry into the lower respiratory tract (LRT), then a second clean bronchoscope was inserted and used to sample the lung by serial bronchoalveolar lavage (BAL). The upper respiratory tract (URT) was also sampled by oral wash (OW). Analysis of the total amount of bacteria present by Q-PCR showed that the amount of bacteria recovered fell sharply during repeated lung sampling, indicating that bacteria detected in early lung samples even with this two-scope procedure mostly derived from oropharyngeal carry-over. In the cleanest samples, low levels of potentially lung-derived bacterial DNA were detected. However, deep sequencing of 16S rDNA tags showed that the bacterial populations present were mostly indistinguishable from URT bacteria in both composition and relative abundance. This suggested that the measured lung microbiota in healthy people are likely derived transiently from oropharyngeal sources, probably by microaspiration 7,8,9 or else as carry over during sampling, rather than existing as discrete communities independently replicating within the lung. No distinctive lung-specific organisms were found to be common among all subjects. A small number of bacterial lineages were found only in lung samples, but most were seen only in one of the 4 samples from each subject and 87% were characterized by single sequence reads and so were of questionable significance. In one of the six subjects, *Tropheryma whipplei* [10], [11] was found in multiple lung samples but not OW or other upper airway samples [5], suggesting that it did constitute an authentic lung resident and indicating that comparison of BAL to OW has the potential to identify autochthonous lung bacteria. Thus our previous study [5] did not detect a shared lung microbiome distinct from that of the upper respiratory tract and common among healthy individuals.

However, in routine clinical practice it will often not be possible to carry out the two-bronchoscope procedure, so here we report the development of analytical methods for use with single bronchoscope samples to detect lung bacteria in deep sequencing data. It is tempting to simply compare the bacterial lineages in OW and BAL, and call those lineages found only in BAL as lung-specific. However, bacteria show a wide range of abundances in OW samples [12], [13], and replicate samples of the same OW fluid will yield different subsets of low-abundance lineages. Because single-scope BAL samples will be substantially admixed with upper respiratory tract bacteria, comparison of single-scope BAL samples to OW samples will likely reveal lineages unique to each, but this by itself does not provide evidence for the presence of lung-specific organisms in BAL due to issues of sparse sampling of rare organisms. Detection can be improved, however, by comparing OW and BAL samples over multiple replicates of each, which helps control for stochastic sampling of low abundance lineages. In addition to lung-unique organisms, clinically important bacteria replicating within the lower respiratory tract may be genuinely present at both sites either because of URT colonization preceeding lung infection or retrograde transport due to coughing, but authentic lung organisms will be present at higher relative abundance in the lung. Abundances determined for any bacteria in single samples will also be subject to stochastic effects of amplification and sequencing.

**Figure 1:**
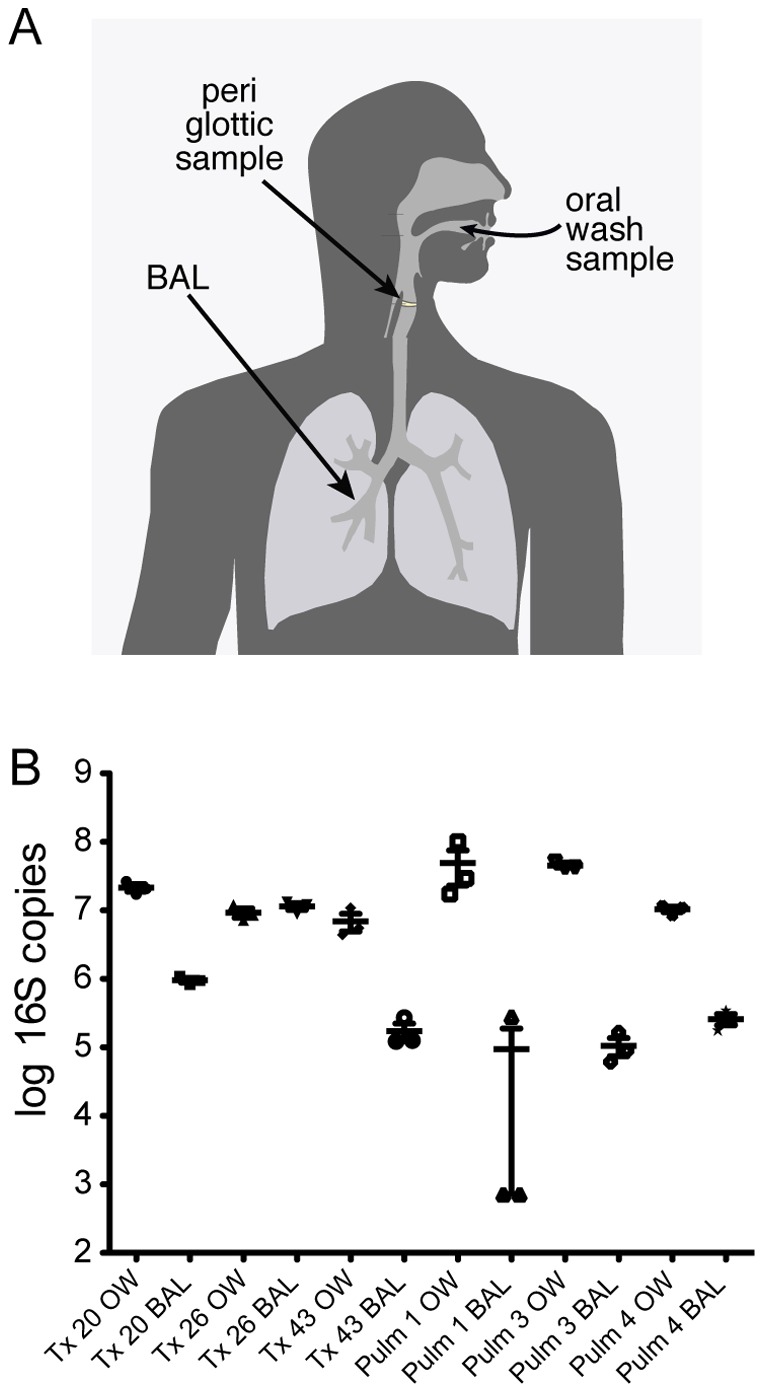
Quantification of bacterial 16S rDNA gene copies in oral wash and bronchoalveolar lavage. A) Diagram of the airway sites sampled in this study. B) The x-axis indicates the sample studied, the y-axis the log of the number of 16S rDNA gene copies estimated by quantitative PCR. Each sample was analyzed in triplicate. Note that this comparison assumes that the average number of 16S rDNA genes per genome is not substantially different for the ensembles of species in each category.

We thus carried out replicate analysis of paired BAL and OW samples from six patients, several with conditions potentially associated with abnormal bacterial populations in the lung, including three lung transplant recipients and one subject each with sarcoidosis, a pulmonary nodule found to be adenocarcinoma, and bronchiolitis obliterans organizing pneumonia (BOOP). Taking advantage of replication, we determined the relative abundance a 16S rDNA lineage must reach to be reliably identified in single samples at different depths of sequencing. We also determined a confidence interval for abundances defined for taxa in single samples. Utilizing the statistical approaches established by this replicate sample analysis, we developed a single-sided outlier test for identifying lineages that are significantly enriched in BAL compared to OW. We then compared these results to data previously derived from healthy subjects sampled at multiple sites (Figure 1A), including both OW and direct sampling from the peri-glottic region [5]. These methods offer a general framework and an analytic pipeline to identify lung-enriched organisms that can be applied to single scope bronchoscopy samples.

## Results

### Experimental design and sequencing

Oral and lung microbiota were sampled from 6 individuals evaluated bronchoscopically for lung disease (Table S1). Three subjects (Tx 20, Tx 26, Tx 43) were lung transplant recipients undergoing routine post-transplant surveillance bronchoscopy, and one subject each had pulmonary infiltrates determined on biopsy and further investigation to be sarcoidosis (Pulm 1), a pulmonary nodule found to be adenocarcinoma (Pulm 3), and pulmonary infiltrates found to be bronchiolitis obliterans organizing pneumonia (BOOP) (Pulm 4). Transplant subjects all had rich histories of exposure to medications, including antibacterial and immunosuppressive drugs, whereas none of the other subjects were on such medications. For each subject, an upper respiratory tract sample was collected by saline oral wash and gargle (OW) prior to transoral bronchoscope insertion. Following routine lower airway inspection, the bronchoscope was wedged in a subsegmental bronchus in the lung allograft for transplant subjects or area of radiologic involvement for the non-transplant subjects, and a saline bronchoalveolar lavage (BAL) sample was obtained. We collected both OW and BAL from each of the 6 subjects for a total of 12 samples. DNA was extracted from three separate aliquots of each of the OW and BAL fluid samples. To assess contamination from instruments, reagents, or environmental sources, sterile water and a saline wash of the bronchoscope channel prior to insertion in the patient were processed alongside biological samples [14], [15]. BAL samples were also submitted for standard microbiological culture of bacterial organisms.

To estimate the total amount of bacteria in each sample, the copy number of bacterial 16S rDNA genes was quantified by Taqman Q-PCR (Figure 1B). For five out of six subjects, the oral cavity contained ∼10 fold higher amounts of 16S rDNA than BAL with an average of 2.4×10^7^ 16S rDNA copies/ml in OW (range 6.9×10^6^–4.9×10^7^) and 2.2×10^6^ copies/ml in BAL (range 9.4×10^4^–1.1×10^7^). Subject Tx 26 had equivalent copies of 16S rDNA in OW and BAL, and had *Pseudomonas aeruginosa* detectable by microbial culture and sequence analysis (below). Most replicate DNA extracts yielded consistent absolute amounts of bacterial DNA, except for Pulm 1, in which BAL replicates 1 and 3 but not 2 were below the lower limit of quantification (725 copies/mL).

Bacterial microbiota were then surveyed by PCR amplification and sequencing of bacterial 16S rDNA genes over the V1–V2 region. Each extracted DNA sample was amplified with a different barcoded primer pair in triplicate reactions, which were combined and subjected to 454 pyrosequencing. Pyrosequencing generated 409,505 raw partial (∼360 bp) 16S rDNA gene sequences, yielding an average of 5,461 reads/sample after quality filtering. Sequence reads were binned at 97% identity for analysis as operational taxonomic units (OTUs, Table S2).

**Figure 2:**
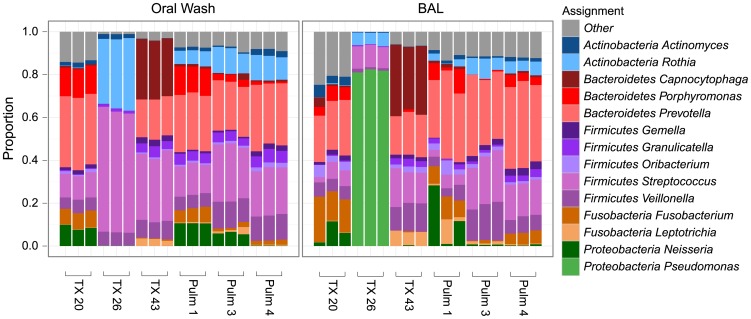
Relative abundances of bacterial genera found in oral wash and BAL samples. The x-axis shows the sample studied, the y-axis the proportion of each lineage as reported by comparison to the RDP database. Taxa are indicated at the Genus level.

The types and relative abundances of bacterial genera comprising each community are summarized in Figure 2. Each column summarizes sequences recovered from a separate DNA sample, grouped by subject ID. The relative abundance of each bacterial genus is shown by the color code. Oral wash contained organisms typical of the oral cavity including a high abundance of *Streptococcus* and *Prevotella* [16], [17], [18], [19]. BAL samples were characterized by similar types of bacteria as in OW, as expected [4], [5], with the exception of subject Tx 26, who was dominated by *Pseudomonas*, which was also found by culture (Table S1). The relative abundance of bacterial organisms was similar among repeat extraction and sequencing of the same biological samples. Pulm 1 BAL replicates varied the most, with the recovery of *Prophyrmonas* and *Neisseria* differing among the extractions, correlating with the observation that this sample had the fewest starting copies of 16S rDNA genes (Figure 1B), and consistent with previous literature that lower starting template copies correlate with greater dispersion among replicates [20], [21].

### Detection of bacterial lineages in airway samples

In some cases, authentic lung bacteria are obvious based on dominance in BAL compared with OW, such as *Pseudomonas* in subject Tx 26 [22], [23]. However, in most cases true lung inhabitants must be identified based on enrichment relative to the upper airway, both because of bronchoscopic carryover and true URT entry into the lung through aspiration [7], [8], [9]. In addition, respiratory pathogens often colonize the URT prior to infecting the lung [24], [25], [26], or may be transported from the lung to the URT by coughing.

Our goals here were 1) to assess the degree to which repeated sampling from single fluid samples can improve the detection of lung-specific bacteria, and 2) to assess the limitation of single-scope single-BAL sampling. One danger in analyzing single OW and single BAL samples is that variation in the detection of low level OTUs will falsely yield OTUs apparently enriched in BAL that are in fact statistical fluctuations in the detection of low-level lineages in OW. Thus, as a first step, we sought to determine the limits of reliable detection of low-level lineages in OW and BAL samples.

**Figure 3:**
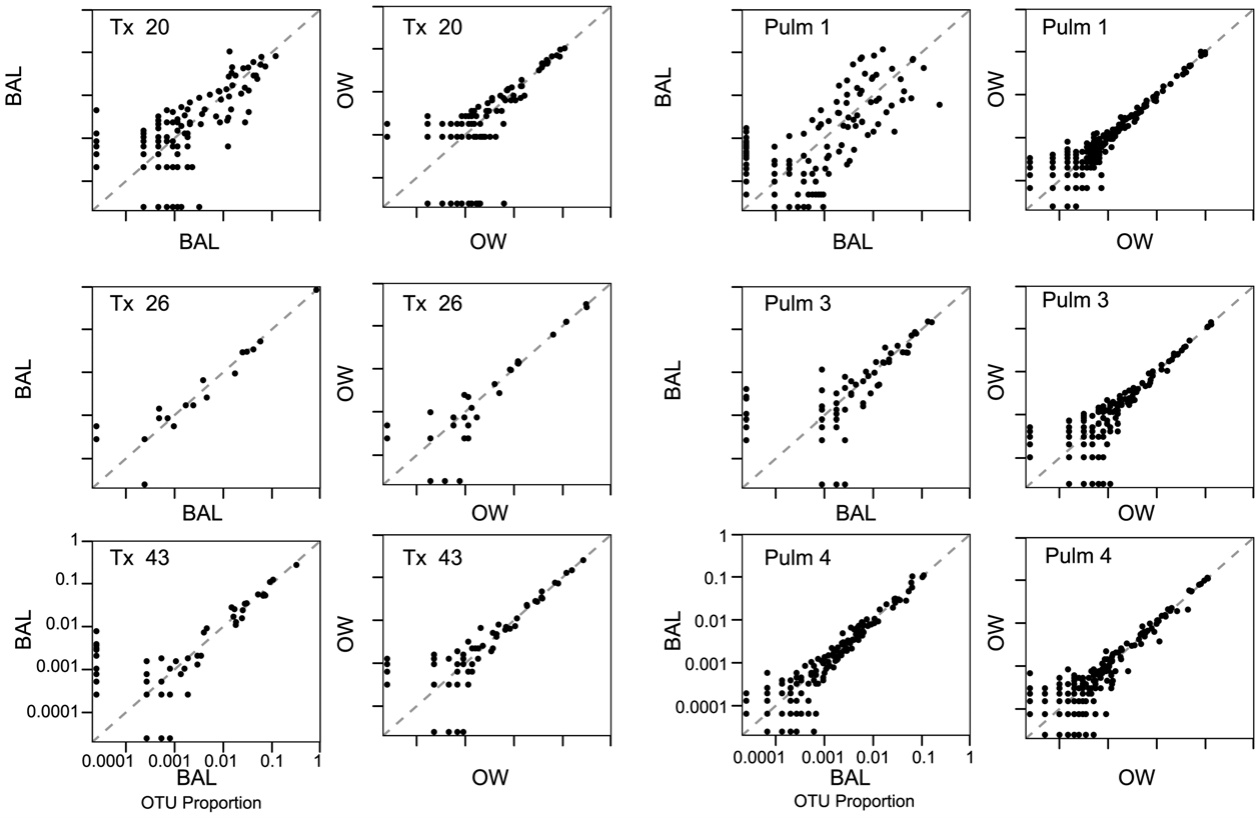
Examples of reproducibility in OTU proportions for replicate samples. Bivariate plots are shown comparing OTU abundance from replicate sequence analysis of aliquots from the same BAL or OW sample. The type of sample is indicated on each axis, the subject of origin inside each box. Proportions are shown on a log scale. The pairs of replicates used for display were chosen randomly from among the three for each sample.

Figure 3 shows bivariate plots comparing OTU abundances in pairs of OW samples or BAL samples from each of the six individuals, where each sample in a pair represents a DNA extraction from different aliquots of the same fluid sample. Each fluid sample was analyzed in triplicate, but for simplicity only one pair-wise comparison is shown for BAL and OW for each subject (the full set is in Figure S1). The proportion of each OTU was plotted as a function of its abundance in each pair of samples. OTUs found only in one sample are found abutting the x or y axes of each plot. The figures reveal good though not perfect agreement between replicates for the most abundant OTUs (upper right in each plot), but less good agreement for more rare OTUs (lower left). The BAL sample from Pulm 1, which showed the lowest number of 16S rDNA copies and the highest degree of Q-PCR variation, showed a relatively high level of variation in OTU proportions.

**Figure 4:**
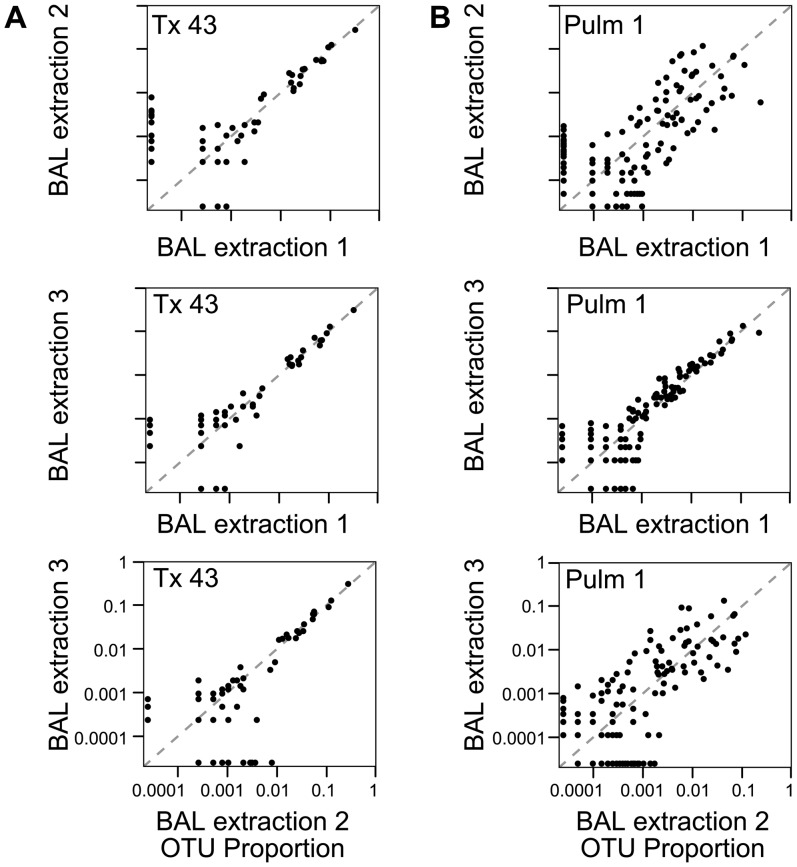
Comparison of reproducibility for all three BAL samples from subjects Tx 43 and Pulm 1. The subject is indicated at the top within each box, the replicates compared are indicated along each axis.

For comparison, Figure 4 shows all pairwise comparisons for Tx 43 and Pulm 1 among the three BAL samples. For Tx 43 (Figure 4A), all replicates showed excellent agreement for high abundance OTUs, though greater variation for low abundance OTUs. Pulm 1 BAL extractions showed more variation (Figure 4B) –while the abundances of OTUs recovered in extractions 1 and 3 were highly concordant, those in extraction 2 were less so. In every comparison, there were OTUs that were found only in one replicate. OTUs unique to each extraction were always infrequent, with no OTU found in a single extraction representing >1% of the total counts.

We therefore determined how abundant an OTU must be in order to be reliably detected upon repeat analysis of the same airway fluid sample. We asked what proportion of the total an OTU must reach to be identified in 95% of replicates at a given sequencing depth. For each set of replicate samples, we modeled the probability of the observed OTU counts, represented as a vector, using the Dirichlet-multinomial distribution [27]. The Dirichlet-multinomial distribution accounts for over-dispersion, or variability of the underlying OTU proportions between observations (see Report S1 for more details). The overall divergence between replicates is best characterized by the quantity *θ*, which is large when replicates are dissimilar, and zero when the variation in OTU counts can be explained by repeat sampling with fixed OTU proportions. An estimate of *θ* was determined for each set of sequencing replicates using the method of moments (Table S3). For the re-extraction replication, the range of *θ* is 0.0002–0.0325, with median 0.0029.

**Figure 5:**
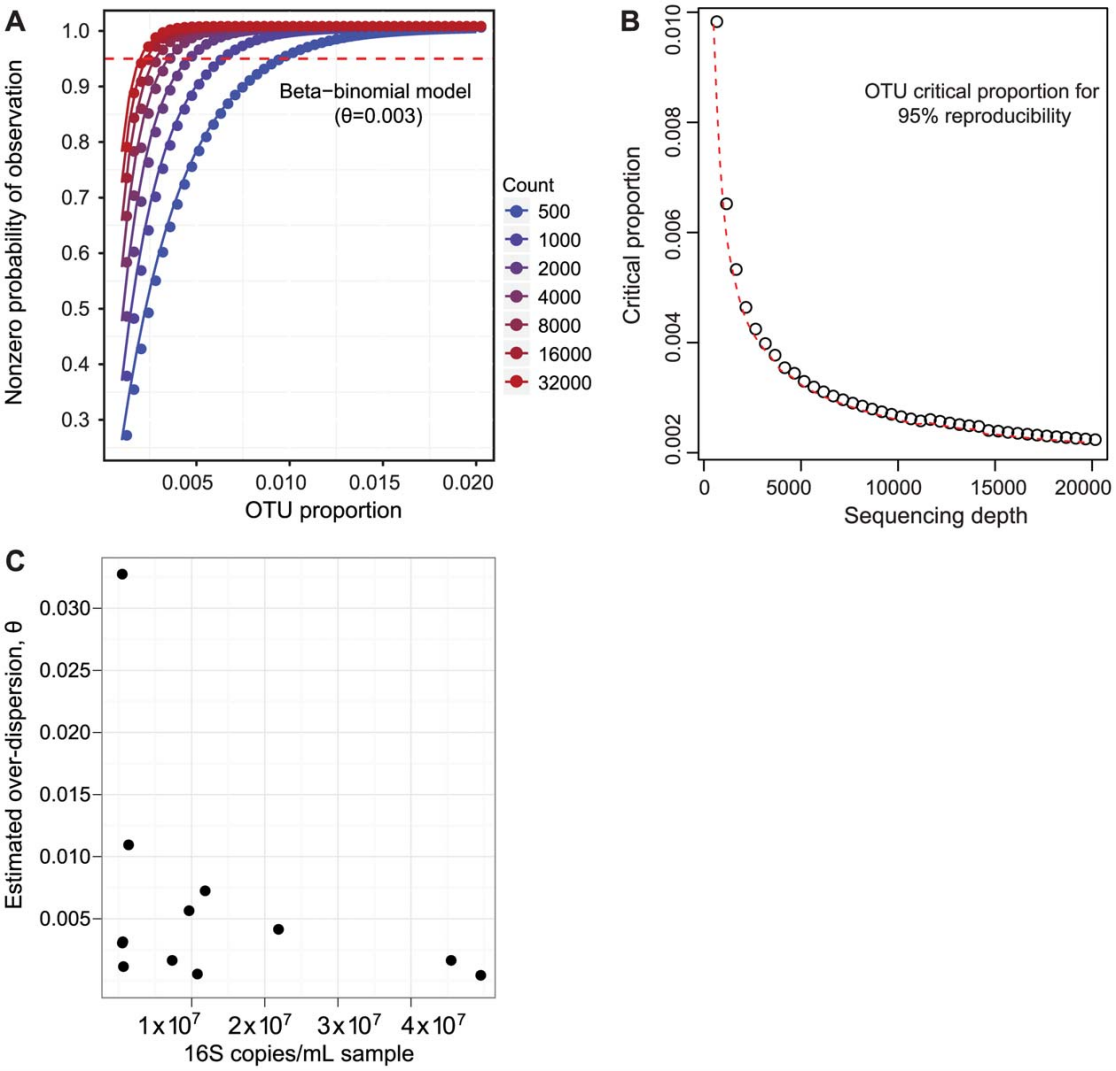
Quantification of sequencing depth necessary for 95% confidence in detection of OTUs at different abundances by different sequencing depths. (A) Probability of observing an OTU upon repeat sequencing of 16S rDNA at a given OTU proportion and sequencing depth, as modeled by a beta-binomial distribution. The level of over-dispersion used in the plot, *θ* = 0.003, is the median estimate from all replicate sample comparisons. (B) Plot of 16S rDNA OTU critical proportion for 95% confidence of detection. (C) 16S rDNA copies/mL in each sample (x-axis) vs. the over-dispersion parameter *θ* (y-axis), indicating that a greater level of over-dispersion is associated with fewer starting copies.

Using the median *θ* value of 0.003, we plotted the probability of a nonzero count against the OTU proportion under different sequencing depths (Figure 5A and B). At a sequencing depth of 500 reads, an OTU must have a proportion of at least 1.0% to be 95% sure that it will be detected in the next extraction and sequencing reaction. For a depth of 5000 reads, the proportion is 0.3%. The critical OTU count at 95% reproducibility was fit to the quadratic form
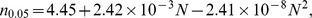
where *N* is the sequencing depth (Figure S2). A polynomial of degree two (quadratic) fits the data best, achieving an R-squared 0.9994. Adding higher degree terms did not improve the fit significantly.

Using the largest value of *θ* derived from our data (0.03) as an estimator of the worst-case scenario instead of the median value (0.003), the critical proportions increase to 3.2% and 1.8% for 500 and 5000 read depths, respectively (Figure S3). From previous work [20], [21], we expected that lower biomass samples would show the greatest variability on re-extraction and sequencing. Figure 5C shows that at low absolute values of 16S rDNA the over-dispersion amongst replicates can range from high to low, while higher biomass samples typically showing lower *θ*.

### Identifying 16S rDNA OTUs enriched in BAL in single comparisons to OW

**Figure 6:**
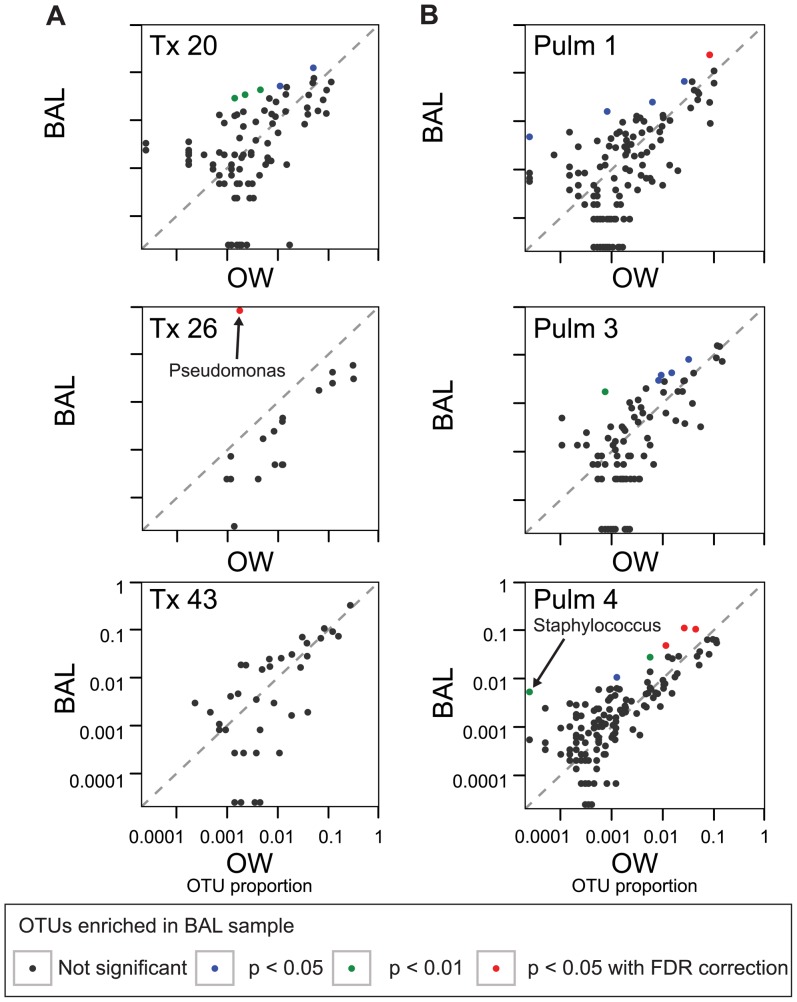
Characterization of lung-specific microorganisms using single sided outlier plots to compare single OW and BAL samples. A single OW sample (x-axis) and a single BAL sample (y-axis) were used for comparison for each subject (out of three samples of each for each subject). The color code indicates the results of significance testing for OTU outliers against a beta-binomial distribution. “FDR” indicates false discovery rate.

The major goal of this study was to identify authentic lung inhabitants by comparing BAL samples to OW samples, thereby allowing us to account for the background arising from oropharyngeal carry-over during bronchoscopy or transient entry of URT bacteria due to aspiration. In this analysis, we assumed that organisms genuinely replicating in lung would for this reason be present at greater relative abundance in BAL. We thus developed a statistical method to distinguish lineages selectively enriched in BAL samples compared to OW. The distribution of abundances in BAL and OW samples was modeled using the Dirichlet-multinomial distribution, as before. Outlier OTUs enriched in the direction of greater representation in lung were identified by a statistically significant departure from the underlying distribution. Several other methods are available for investigating lineages differing between microbiome samples (see [27], [28], [29] and references therein), but none are optimized for identifying outliers in pairs of samples with single replicates of each (See Report S1 for discussion). Figure 6 shows pairwise analysis for single samples for each subject by plotting the abundance of each OTU in OW on the X axis and in BAL on the Y axis, where representative replicates were chosen from among the three available for each BAL and OW (the full set of pairwise comparisons is in Figure S4).

The extent of enrichment of outlier lineages in lung is shown by the color code. Lineages that were significantly enriched after correction for multiple comparisons, the ones of main interest, are shown in red. Significantly enriched outliers could be seen in three of the six samples (Tx 26, Pulm 1, Pulm 4), in the replicates chosen for illustration in Figure 6.

**Figure 7:**
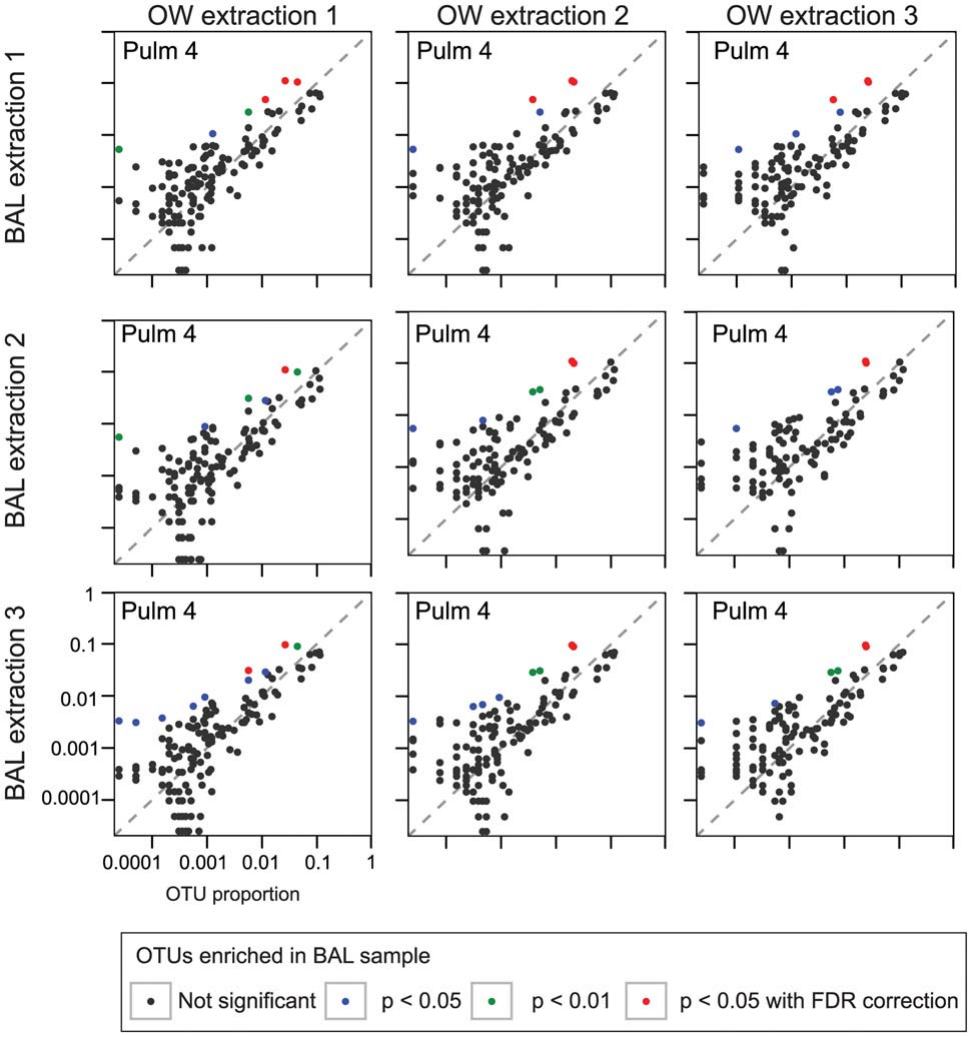
Characterization of all pair-wise comparisons of the three BAL and OW samples from Pulm 4. The three OW samples are shown on the x-axis, the BAL samples on the y-axis. The significance is again shown by the color code indicated at the bottom of the figure.

Figure 7 shows all pairwise comparisons for Pulm 4, the subject with BOOP. Each of the three OW samples is compared individually to each of the three BAL samples, for a total of nine comparisons. One OTU, corresponding to *Prevotella*, is amongst the most abundant lineages in both OW and BAL, and is significantly enriched in the BAL samples in all nine comparisons. The adjacent OTU, also assigned to *Prevotella* is significantly enriched in seven of the nine comparisons. In the remaining two comparisons, this lineage was statistically enriched, but at a level that did not retain significance after correction for multiple comparisons. In a possible parallel, *Prevotella* has been suggested to be enriched in certain diseased oral samples [30]. Three other lineages were also enriched in all nine comparisons (Table 1), including *Staphylococcus*, which was also found by BAL culture (below), but not at a level that survived correction for multiple comparisons. These findings show that lung-enriched organisms can be identified in some comparisons, but emphasizes that pairwise comparisons of single samples do not always yield identical results.

**Table 1:** Bacterial lineages enriched in BAL samples identified after replicate sampling of each subject.

Sample ID	OTU ID	Taxonomic assignment	P-value in combined outlier analysis (FDR adjusted)	Mean BAL porportion	Mean OW porportion	Fold change	# times called as outlier in pairwise comparisions (FDR adjusted, max = 9)	# times called as outlier in pairwise comparisions (unadjusted, max = 9)	# additional OTUs called in pairwise comparisions (unadjusted)
**Tx 20**	236	*Fusobacterium*	0.0024	0.036	0.004	8.9	2	7	5
	2438	*Campylobacter*	0.0207	0.015	0.001	13.9	0	3	
	1109	*Oribacterium*	0.0370	0.037	0.010	3.6	0	6	
	2163	*Fusobacterium*	0.0437	0.095	0.051	1.9	0	5	
**Tx 26**	517	*Pseudomonas*	<0.0001	0.819	0.001	580.9	9	9	0
**Tx 43**	None	NA	None	NA	NA	NA	None	None	2
**Pulm 1**	214	*Prevotella*	<0.0001	0.037	0.003	11.1	0	5	6
	1032	*Veillonellaceae spp.*	<0.0001	0.052	0.001	59.6	3	9	
	1653	*Lachnospiraceae spp.*	<0.0001	0.030	2.51E-04	120.7	3	6	
	333	*Leptotrichia*	0.0017	0.030	0.002	19.6	0	6	
	2074	*Neisseria*	0.0051	0.110	0.082	1.3	3	3	
	1284	*Leptotrichiaceae spp.*	0.0073	0.020	0.001	36.8	0	3	
**Pulm 3**	214	*Prevotella*	<0.0001	0.039	0.008	5.0	0	9	5
	1032	*Veillonellaceae spp.*	<0.0001	0.020	0.001	34.4	0	9	
	1271	*Prevotella*	0.0006	0.051	0.014	3.7	0	9	
	338	*Streptococcus*	0.0009	0.078	0.030	2.6	0	9	
	1908	*Selenomonas*	0.0015	0.011	1.81E-04	59.9	0	7	
**Pulm 4**	2151	*Prevotella*	<0.0001	0.097	0.030	3.3	7	9	7
	2156	*Porphyromonas*	<0.0001	0.035	0.007	5.0	3	9	
	2207	*Prevotella*	<0.0001	0.104	0.023	4.5	9	9	
	2428	*Fusobacterium*	<0.0001	0.030	0.006	4.9	1	9	
	1355	*Staphylococcus*	0.0144	0.005	3.58E-05	130.2	0	9	

OTUs were identified as significant outliers after controlling for a false discovery rate (FDR) of 5%. The same OTUs were rarely identified in pairwise comparisons when controlling for an FDR of 5% (column 8). When no FDR adjustment was made, the OTUs were often identified in pairwise comparisons (p<0.05, column 9). Under these circumstances, however, several additional OTUs were identified in pairwise comparisons that did not achieve significance in the analysis of replicate sampling (column 10).

### Identifying 16S rDNA OTUs enriched in BAL, taking advantage of replication

Table 1 summarizes the lineages over all six subjects that were significantly enriched in BAL, taking advantage of the three replicates of each. Only those values are shown that were significant after correction for multiple comparisons, which controlled for a false discovery rate (FDR) of 5% (see methods). The number of significantly enriched lineages varied from zero for one of the transplant subject (Tx 43), to six for the sarcoidosis subject (Pulm 1). Up to seven additional lineages were called as significantly enriched in lung in single pair-wise comparisons in each subject but were not confirmed in the replicated data, emphasizing that comparisons between single samples will sometimes yield misleading conclusions. Assuming the replicate analysis better approximates the underlying distribution, we conclude that replication both helps minimize incorrect calls while helping distinguish correct calls. We return to these points in the Discussion.

### Comparison to results from culture-based analysis

BAL samples from the six subjects were also analyzed by conventional bacterial culture and three were positive for culturable pulmonary pathogens (Table S1). BAL from one lung transplant subject (Tx 26) was positive for *Pseudomonas aeruginosa*. Two BAL samples were positive for *Staphylococcus aureus*–one from transplant subject Tx 20, and the other from the BOOP subject Pulm 4. The *Pseudomonas* infection and one of the *Staphylococcus aureus* infections (Pulm 4) were identified as enriched in lung in the pooled sequence analysis (Table 1). The *Pseudomonas* OTU of Tx 26 was called as significantly enriched (p<0.05 after correction for multiple comparisons) in all pairwise comparisons and also in the combined outlier analysis. This OTU was enriched 581-fold in BAL relative to OW, the greatest extent of enrichment seen in this study, and comprised 82% of the lung sequences. The *Staphylococcus* OTU of Pulm 4 was called as an outlier in all nine pairwise comparisons, and at p<0.05 after correction for multiple comparisons in the pooled analysis. However, this OTU was relatively low in abundance (0.5%) in BAL, and did not reach significance in any pairwise comparison after correction for multiple comparisons. This OTU was enriched 130-fold, the second greatest enrichment seen in BAL. In contrast, for Tx 20, *Staphylococcus* was detected in the sequencing data, but was present at a low level and was not significantly enriched in lung, suggesting that culture resulted in outgrowth of this organism.

The majority of lung-enriched organisms detected in BAL by sequencing (Table 1) are recognized URT inhabitants and/or anaerobes and not typically targeted by respiratory tract culture techniques.

### Comparison of lung and upper respiratory tract samples from healthy subjects

**Figure 8:**
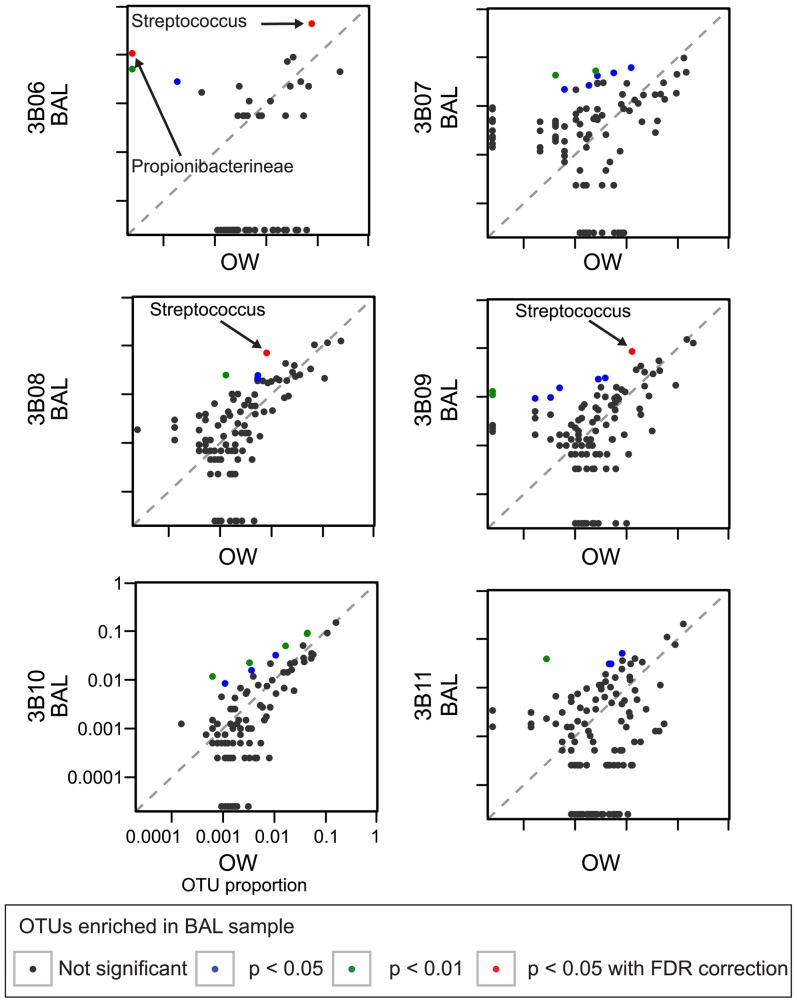
Application of the single sided outlier analysis to a single OW and BAL sample from a healthy population. An OW sample (x-axis) and the BAL 1^st^ Return sample (y-axis) were used for comparison for each subject. The color code indicates the results of significance testing for outliers against a beta-binomial distribution. Data from [5].

We then asked if our outlier detection method would identify outliers as significantly enriched in a single pair-wise comparison of BAL and OW from 6 healthy individuals studied in depth previously using a two-bronchoscope procedure [5], where the BAL and OW pairs were found to be closely similar. To be most comparable with clinical single-scope bronchoscopy, the BAL first return was compared with OW samples. As was discussed above, lung-restricted lineages were detected only rarely in the previous analysis of these subjects. Here we used the single sided outlier method to investigate lung enrichment, and found that three of the six subjects (3B06, 3B08, and 3B09) had potential lung-enriched OTUs in BAL compared to OW after correction for multiple comparisons (Figure 8).

For subject 3B06, *Streptococcus* and *Propionibacterineae* OTUs were identified as lung-enriched outliers. However, this BAL sample was notable for having the lowest number of 16S rDNA copies by Q-PCR of the six, and the apparent lung-enriched lineages were abundant in controls such as saline pre-wash of the bronchoscope channel [5]. This result suggests that the source of these DNA sequences was environmental rather than lung.

Subjects 3B08 and 3B09 each had one *Streptococcus* OTU called as a significant outlier in BAL compared with OW out of a total of 194 and 217 BAL OTUs for each. We asked whether these taxa would remain significantly enriched in lung if instead of OW, we compared BAL to peri-glottic bacteria on the tip of a bronchoscope that reached the glottis but did not enter the lower respiratory tract (Scope 1 Tip). As shown in Figure S5, these taxa were not significant outliers when compared with the peri-glottic Scope 1 Tip samples. Thus, the apparent lung-enrichment in these two BALs likely results from OW providing an imperfect surrogate for bacterial species that either contaminate a bronchoscope passing through the peri-glottic region, or alternatively that may be present in the lung due to passive aspiration of peri-glottic material.

## Discussion

Microbiological sampling of the lower respiratory tract is a challenge due to URT admixture through bronchoscopic carry-over [1], [2], [3], [4], [5]. Bacteria may also be present in the lung due to passive entry by microaspiration of upper airway microbes without authentic replication in the lung. Characterization is further confounded by the fact that true lung inhabitants of clinical importance may be present in the URT as well, either because of URT colonization prior to lung infection [24], [25], [26], or retrograde movement due to coughing. There are typically much greater densities of bacteria in the oral cavity than in lung [2], [31], [32], [33], [34], meaning that lung bacteria in BAL may represent a minority of lineages in the sample, further complicating detection. Stringent sampling of lung is possible in research settings, for example using a two-bronchoscope procedure or endotracheal intubation [5], [35], but this will not be feasible in most clinical settings. Here we present analytical methods for identifying bacteria selectively enriched in lung and their application to six clinical specimens chosen to represent a range of community profiles. An assumption underlying this model is that carry-over from URT into lung (or entry by aspiration without replication) would result in proportional abundance in lung no different from that in URT samples. Authentic lung inhabitants that are replicating in the lower respiratory tract, in contrast, would be enriched in BAL compared with the URT. In this study, we used replicate extractions of BAL and matched OW fluid to assess analytical approaches and improve sensitivity. We first defined the confidence with which any given taxon can be identified and quantified in a particular respiratory tract sample. We then constructed a single-sided outlier test to identify the significantly enriched taxa in BAL compared with OW, taking account of the uncertainties in quantification. To make the analysis more feasible, we studied clinical conditions where lung microbial populations are expected to be abundant, such as after lung transplant.

It is tempting to compare one BAL sample to one OW, identify lineages selectively present in the BAL, and conclude that these represent lung-specific organisms. However, OW communities contain a mixture of abundant and rare organisms, so that in a typical collection of 16S rDNA pyrosequence tags, most of the abundant lineages will be represented, but only a subset of the rare lineages will be present. Comparison of two OW samples from the same individual will yield mostly the same abundant lineages, but only partially overlapping subsets of rare lineages, as can be seen in Figure 3. Thus comparison of a single OW sample to a single BAL sample from the same individual–where the BAL sample contains substantial oropharyngeal bacteria–will likely show some divergence in the rare lineages, but calling these as lung-enriched would often be erroneous. To strengthen the assignment of lineages enriched in BAL to lung, we have investigated the use of replicate sampling.

We first carried out a simple analysis of the relationship between sequencing depth and the likelihood of identifying an OTU present in a given proportion in the sequence population. We found that at a typical sequencing depth of 5000 tags, OTUs of proportion 0.3% or greater could be called with 95% confidence. Figure 5A and B presents a generalization of this over many sampling depths. Thus, calling lung-specific lineages is only possible where the proportions are above a critical sample-specific threshold determined by the depth of sequencing.

We then developed a statistical method for identifying bacteria enriched in lung. The population frequency distribution was modeled using the Dirichlet-multinomial distribution, and outliers in the direction of lung-enrichment identified. The results are somewhat sobering. In the case of one lung transplant recipient (Tx 26), *Pseudomonas aeruginosa* was detected at high levels in BAL samples (∼82%). *Pseudomonas* was called as significantly enriched in BAL in all comparisons between OW and BAL samples, as well as in the pooled analysis of replicates, with an enrichment in BAL of ∼580-fold. This organism was also identified by culture from BAL. However, for the other four subjects where outliers were identified in the replicate data, many of these OTUs were called in only a subset of pairwise comparisons, and in some cases OTUs were called in single pair-wise comparisons that were not substantiated in the replicate analysis (Table 1). Two subjects had a *Staphylococcus aureus* infection by clinical culture, but only one of these was enriched in lung (130-fold) and called as significantly enriched in BAL in our data. These comparisons emphasize that not all organisms detected by culture are enriched in lung.

The analytical methods were also tested on a previously published set of samples from healthy controls [5], providing benchmarks for comparison. The simplest outcome would have been for BAL samples from healthy subjects to show no significant difference from OW, indicating no enrichment of bacteria in lung. For three of the six subjects this was the result. The other three subjects, however, showed a low number of OTUs with significant enrichment in BAL compared with OW, though these were a small minority of all OTUs detected. For one subject, the numbers of total 16S rDNA copies in the BAL sample was low, and the enriched OTUs matched taxa in bronchoscope pre-wash samples and reagent-controls, consistent with the apparent lung-enriched OTUs deriving from contaminants. However, for the other two subjects, the origin of the lineages found enriched in lung compared with OW is more complicated. The absolute proportions of these taxa in BAL were low (<1 to ∼8%) or proportional enrichment was relatively modest (<10-fold). We found that these OTUs no longer scored as enriched when BAL samples were compared to peri-glottic samples (Scope 1 Tip) instead of OW. This suggests that OW may not fully reflect the microbiota carried over by a bronchoscope passing through the URT before entering the lungs or that may enter the lung through normal microaspiration. Thus, OW appears to be a reasonable but not perfect surrogate for bacteria in the URT that may confound bronchoscopic lung sampling. Other possible contributors include inhomogeneity in BAL and OW samples, and imperfect control of over-dispersion and multiple comparisons in the statistics. Thus, for interpreting lung enrichment in clinical samples, it would be most convincing to see higher proportions and greater levels of enrichment than those seen for these two samples. These uncertainties emphasize the challenges of identifying lung-enriched lineages against the background of abundant URT-derived microbiota. Use of these methods to study a large set of lung transplant samples will be reported elsewhere (Charlson et al., submitted).

Based on this study and results in [5], we can suggest the following principles for identifying organisms selectively present in BAL. 1) The URT is heavily colonized with bacteria, so that any sampling strategy needs to take into account carry-over from URT into BAL. Where possible, use of sampling procedures that minimize URT carry-over during bronchoscopy is optimal [5], [35], [36]. 2) Collection of URT samples can enable comparative analysis to specify lung-enriched lineages in BAL, with OW providing a reasonable but not perfect URT sample. 3) Work up of bronchoscope pre-wash and lavage saline controls through the DNA purification procedure in parallel with airway samples can allow identification of sequences originating from instruments, reagents and other environmental sources. 4) Detection of any given taxa is a function of both the proportional abundance of taxa and sequencing depth as described in Figure 5A and B. 5) Over-dispersion among samples increases with low numbers of 16S rDNA copies (Figure 5C), as is well known from older literature [20], [21]. 6) Optimal detection of low abundance taxa is afforded by replicate analysis of BAL and OW samples, though even there the ability to distinguish true lung inhabitants is limited. Software for the statistical methods used in this study is available at http://github.com/kylebittinger/polyafit.

## Methods

### Subjects and sample collection

Specimens were collected from patients undergoing clinical bronchoscopy between September 2010 and December 2011. Prior to bronchoscopy, a 10 mL saline oral wash was collected by brief swish and gargle. A bronchoscope pre-wash was collected by instilling and then aspirating 10 mLs of saline through the instrument channel before insertion into the patient. After nebulized and topical oropharyngeal anesthesia, the bronchoscope was inserted transorally, advanced through the vocal cords, and visual inspection carried out per standard clinical protocol. Bronchoalveolar lavage (BAL) was obtained by instilling and then aspirating 60 to 120 mL saline through the bronchoscope while wedged in the region of interest. Any additional procedures such as biopsies or brushings were done after BAL was completed. BAL was separately partitioned for standard microbial culture and 16S rDNA analysis. Sampling of healthy volunteers with a two-bronchoscope procedure, in which one scope was inserted only to the glottis and a second clean bronchoscope then sampled the lower respiratory tract, has been described in detail previously [5]. To best approximate a single-scope clinical bronchoscopy, the first BAL return was used from those samples for comparison here with OW. Written informed consent was obtained from all subjects under protocols approved by the University of Pennsylvania IRB.

### DNA extraction

DNA was isolated from unfractionated OW and BAL using the PowerSoil DNA isolation kit (MoBio, Carlsbad CA) in a BSL 2+ hood after treatment with DNAOff (MP Biomedicals, Solon OH) and 30 minutes of UV irradiation. 1.8 ml was centrifuged at 10,000×g for 10 minutes at 4°C, pellets resuspended in 60 ul Solution C1 (PowerSoil manufacturer's 1^st^ lysis solution), and transferred to bead tubes. Tubes were incubated at 65°C for 10 minutes, beadbeat for 2 minutes using Minibeadbeater-16 (BioSpec Products, Bartlesville, OK), and extracted per manufacturer protocol. Extracted DNA was stored at −20°C.

### Bacterial 16S rDNA gene PCR amplification, pyrosequencing and sequence analysis

We amplified bacterial 16S rDNA genes using barcoded broad-range V1V2 primers described in [16]. Triplicate 25 ul reactions were performed with AccuPrime Taq DNA Polymerase High Fidelity (Invitrogen, Carlsbad CA), pooled and purified, and pyrosequenced using primer A on a 454 Life Sciences FLX instrument as in [16]. Reads were denoised at the flowgram level with Denoiser [37], [38], and integrated into the QIIME analysis pipeline [28] where they were clustered into OTUs at 97% sequence identity with UCLUST [39]. OTUs were aligned to full-length 16S rDNA sequences with PyNAST [40], and removed if unalignable (minimum 75% identity, 150 nucleotides) or tagged as chimeric by ChimeraSlayer [41]. Sequences were assigned taxonomy with RDP Classifier (50% confidence threshold) [42], and a phylogenetic tree generated *de novo* with FastTree2 [43]. All sequences are deposited at the Sequence Read Archive under accession numbers SRA057171.

### Statistical methods

We use a beta-binomial distribution to model the count of a single OTU in each sample. The beta-binomial distribution is a generalization of the binomial distribution that allows for over-dispersion, or uncertainty in the underlying OTU proportions. This distribution has the form
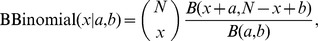
where *x* is the number of reads assigned to the OTU, *N* is the total number of reads, and *B* is the beta function. The parameters, *a* and *b*, control the expected value of OTU abundance, *a*/(*a*+*b*), and the degree of over-dispersion, *θ* = (*a*+*b*+1)^−1^.

If a common value of *θ* is used for all OTUs, the counts can be jointly modeled by a Dirichlet-multinomial distribution, the multivariate version of the beta-multinomial distribution (41). Under this joint model, *θ* is estimated for each set of sequencing replicates (grouped by subject and sampling location) using the method of moments [44]. For a given *θ* and sequencing depth, the critical OTU proportion, defined as the OTU proportion with which the probability of nonzero count is 0.95, is calculated based on the beta-binomial model.

To detect OTUs enriched in BAL samples relative to OW, we again use a Dirichlet-multinomial distribution. A maximum-likelihood estimate of the parameters is determined by numerical optimization. For each OTU, the Dirichlet-multinomial parameter estimates are used to construct a marginal beta-binomial distribution. The marginal form represents the distribution of OTU counts under the null hypothesis that a particular OTU is not enriched in the BAL sample. The p-values reported for BAL enrichment are generated from a one-sided test against the null distribution. This method is conservative, because genuine BAL-enriched OTUs will artificially increase the estimated level of over-dispersion, and thus raise the bar for detection.

For combined BAL-OW samples, the parameters of a Dirichlet-multinomial distribution were estimated as before, treating all BAL and OW samples as separate observations. For each OTU, we summarized the amount detected in BAL samples by computing the mean proportion. A null distribution was generated by sampling repeatedly from the marginal distribution for the OTU in question, and computing the mean proportion for 10,000 trials. One-sided p-values are reported for each OTU.

Correction for multiple comparisons was carried out by adjusting p-values to control for a desired false discovery rate (FDR). An FDR of 5% was used to identify OTUs that were significantly enriched.

## Supporting Information

Figure S1 **Complete set of bivariate plots demonstrating OTU reproducibility for each replicate and all subjects.**
(PDF)

Figure S2 **Fit of the empirical function to calculate the critical OTU count at various sequencing depths.**
(PDF)

Figure S3 **Non-zero probability of observing an OTU upon repeat sequencing 16S rDNA under the worst-case scenario.**
(PDF)

Figure S4 **Complete set of outlier plots for pairwise BAL-OW comparisons.**
(PDF)

Figure S5 **Healthy patient 3B08 and 3B09 outlier plots.**
(PDF)

Table S1 **Summary of subjects and samples studied.**
(PDF)

Table S2 **16S rDNA OTU table.**
(PDF)

Table S3 **Values of** ***θ*** **and standard error for each sample estimated from the beta-binomial distribution model.**
(PDF)

Report S1 **Comparison of the single-sided outlier to other methods of community analysis.**
(PDF)
