# Supplementary figures and images for: Assessing Bacterial Populations in the Lung by Replicate Analysis of Samples from the Upper and Lower Respiratory Tracts
Source: PLoS One. 2012 Sep 6;7(9):e42786. doi: 10.1371/journal.pone.0042786 (PMC3435383; doi:10.1371/journal.pone.0042786)

Extraction 1 vs. 2

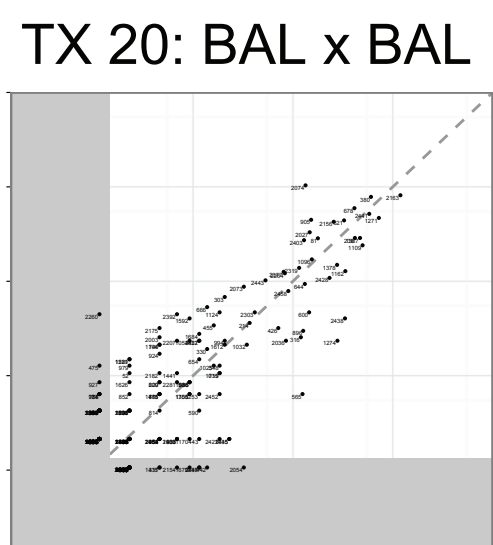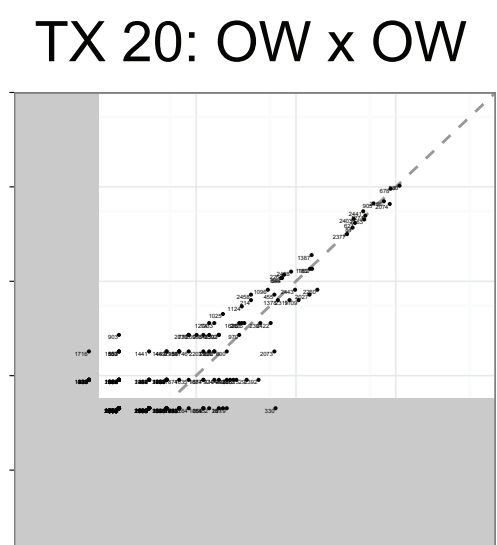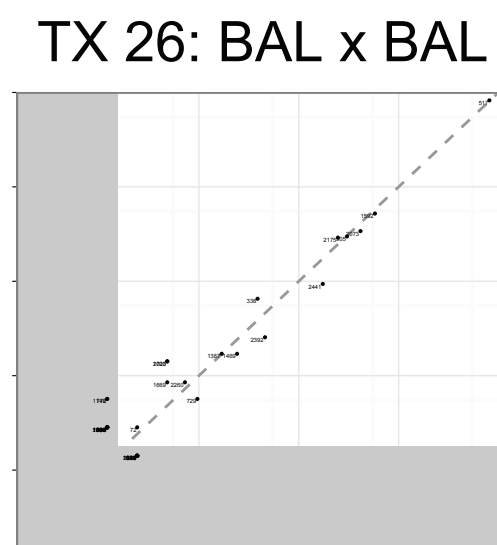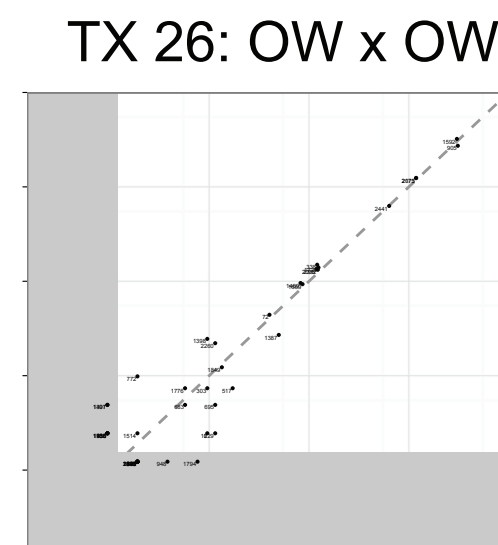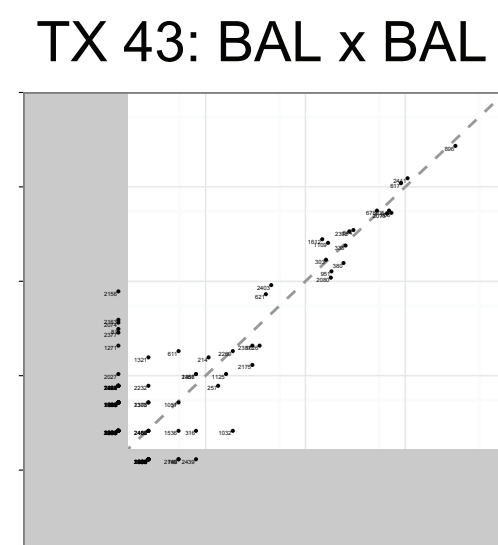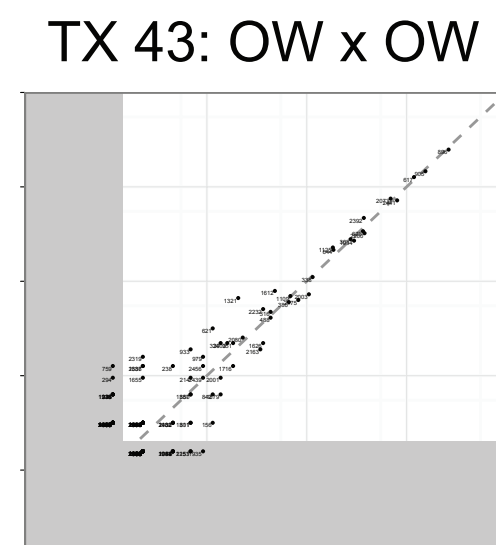

Extraction 1 vs. 3

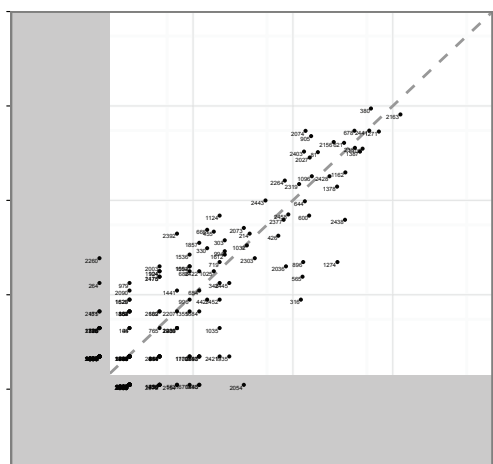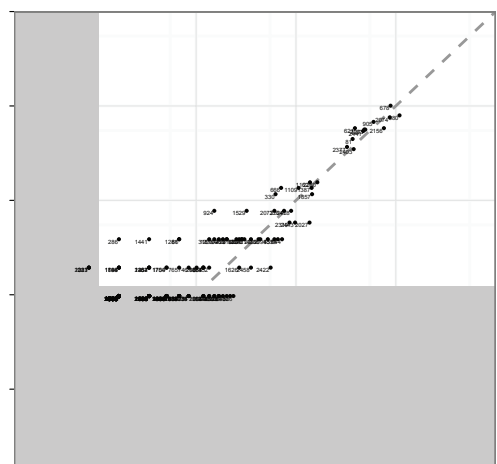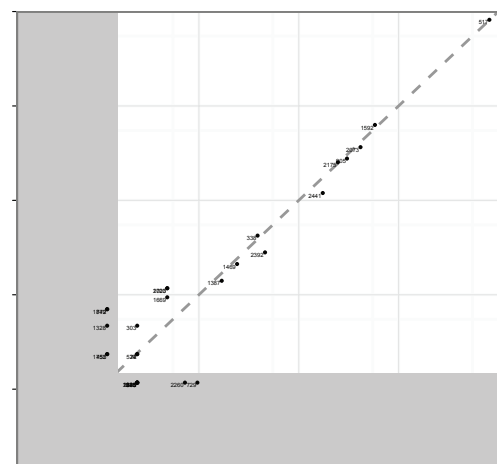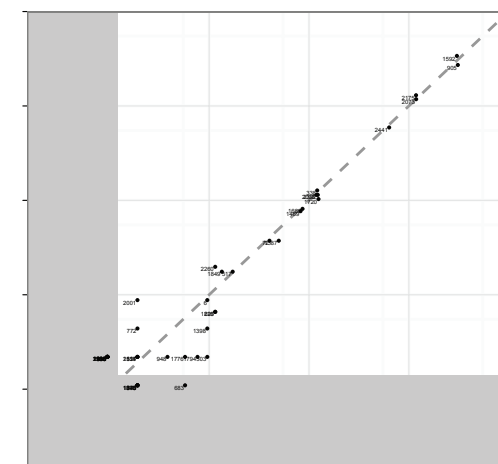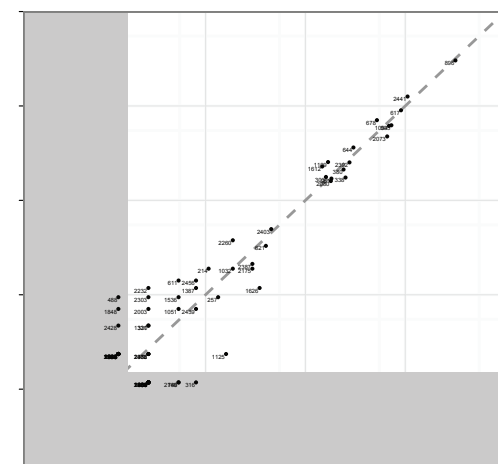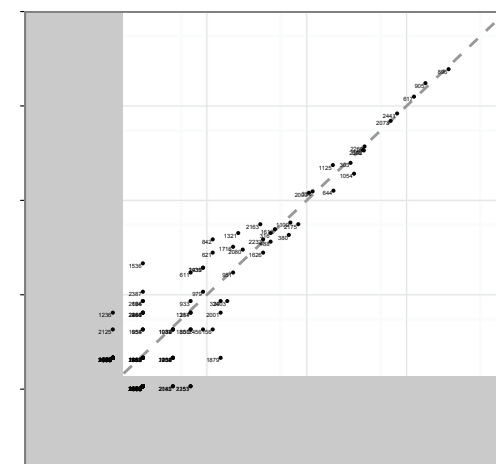

Extraction 2 vs. 3

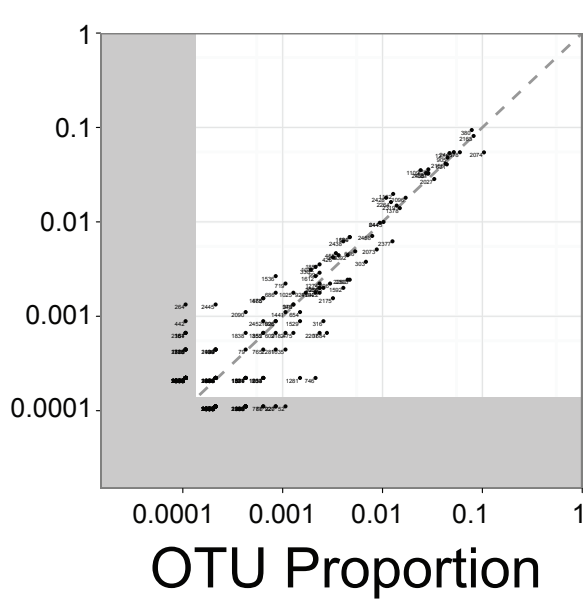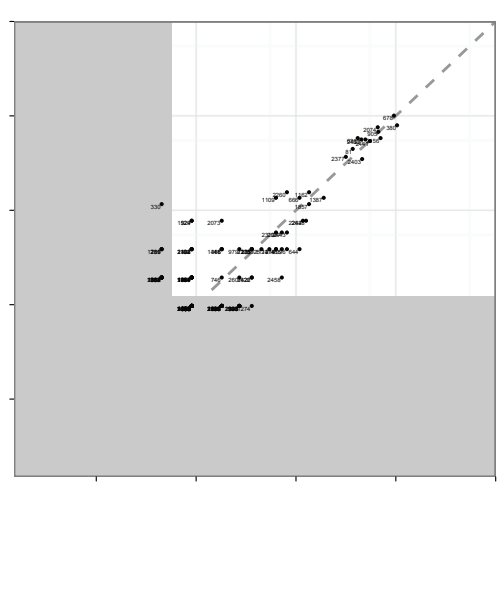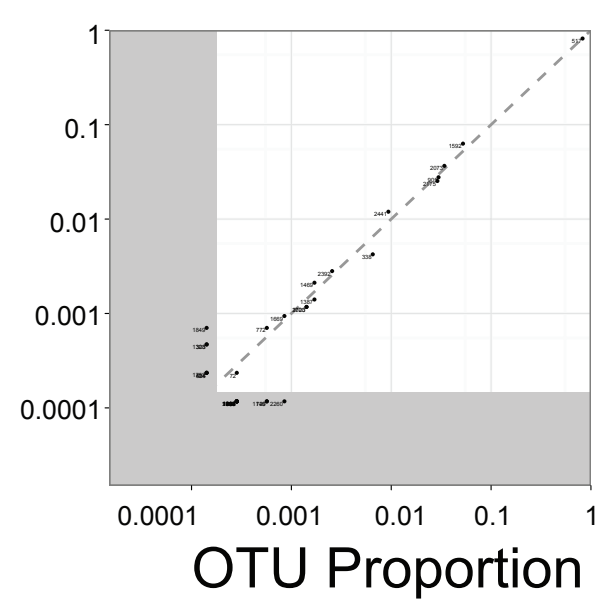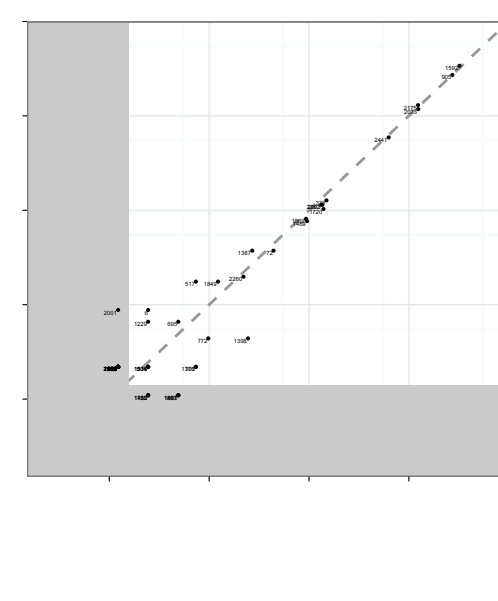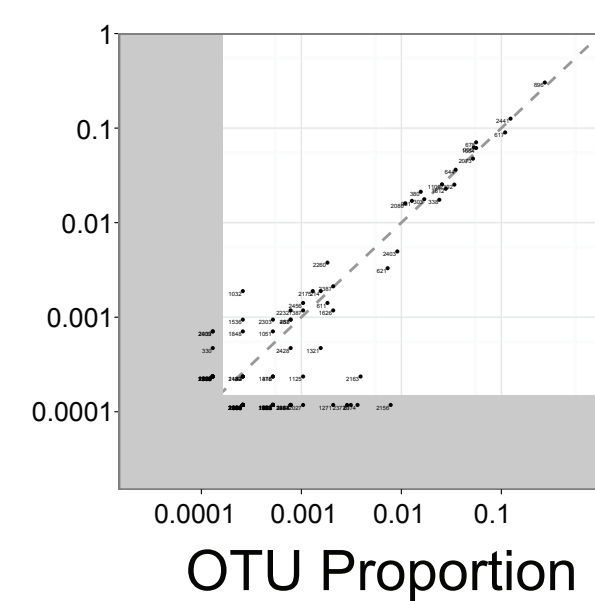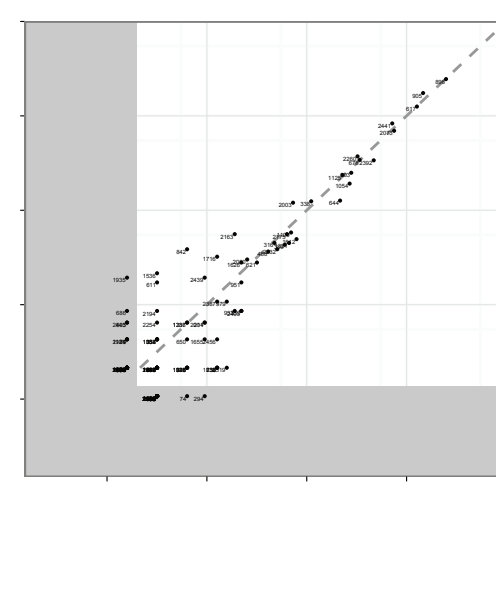

Extraction 1 vs. 2

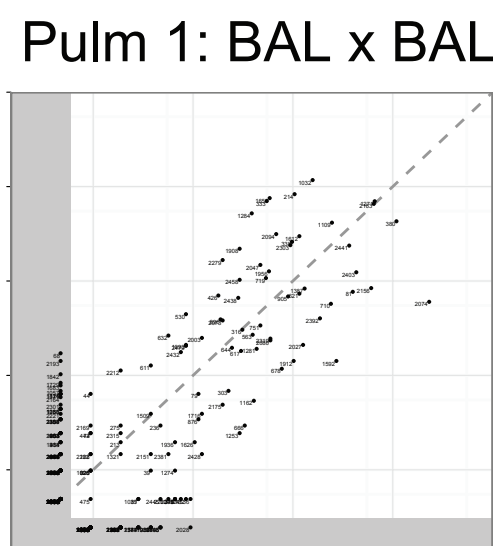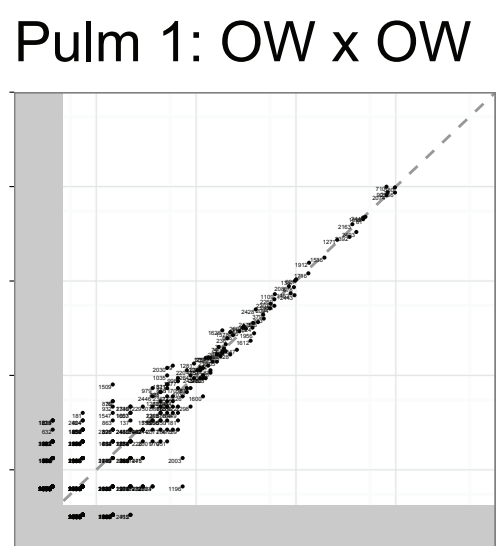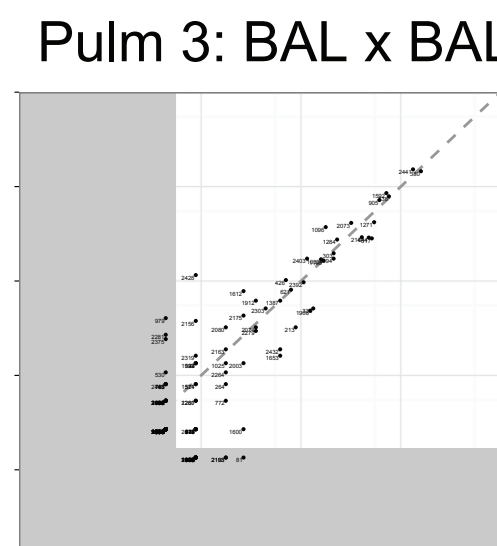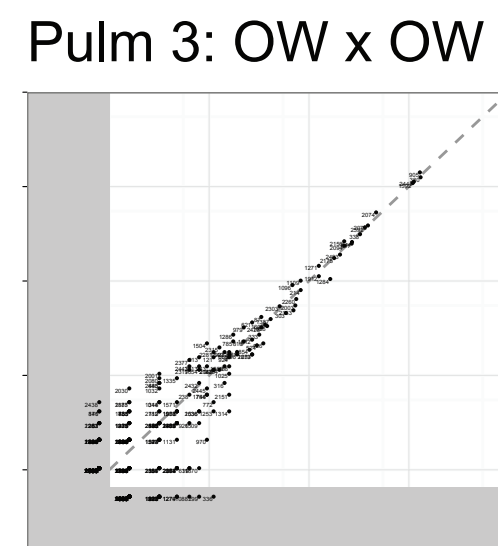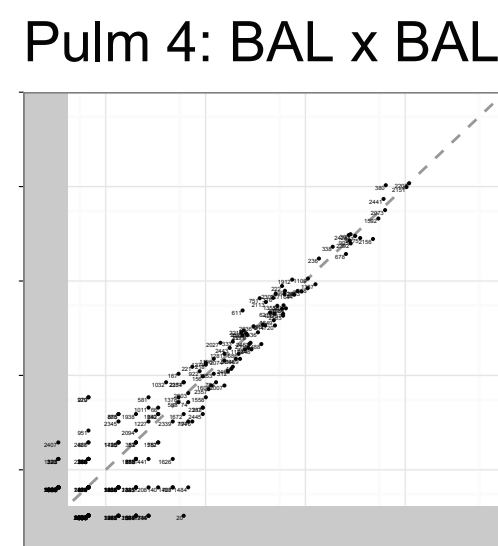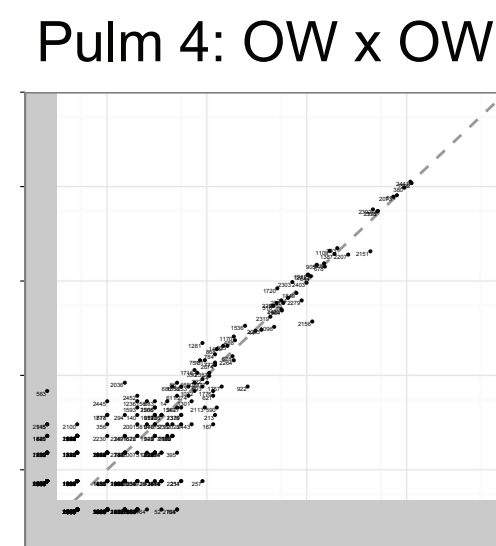

Extraction 1 vs. 3

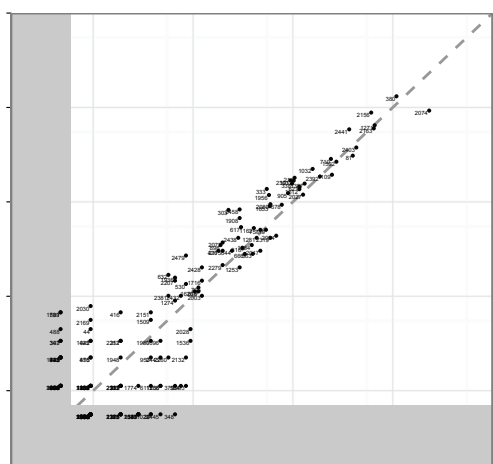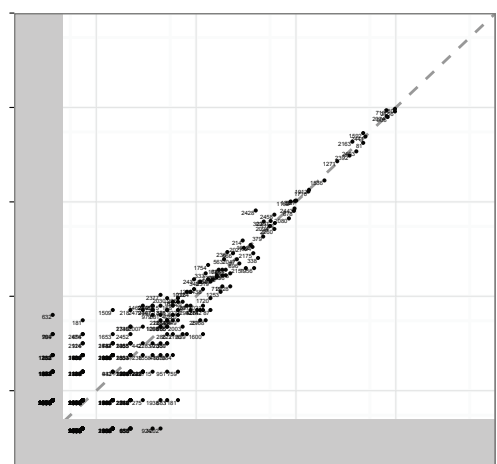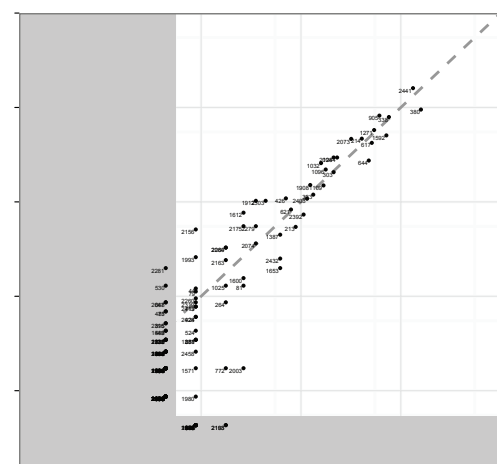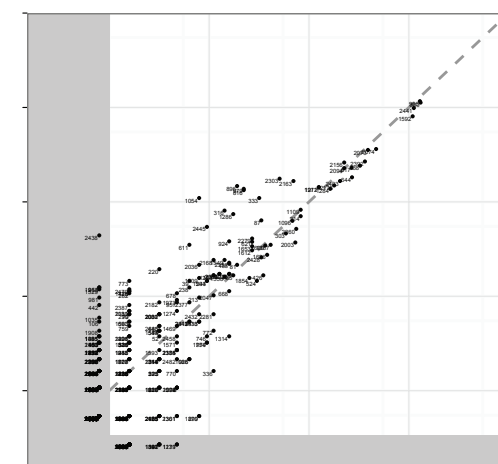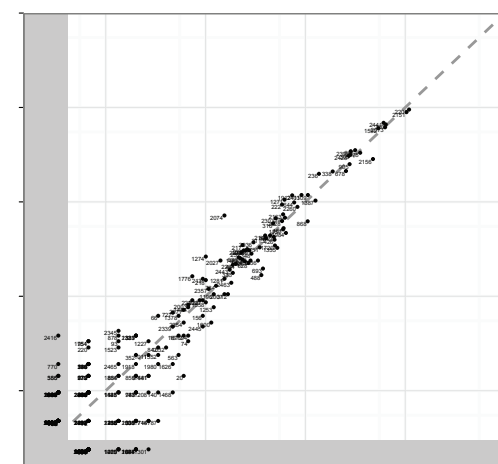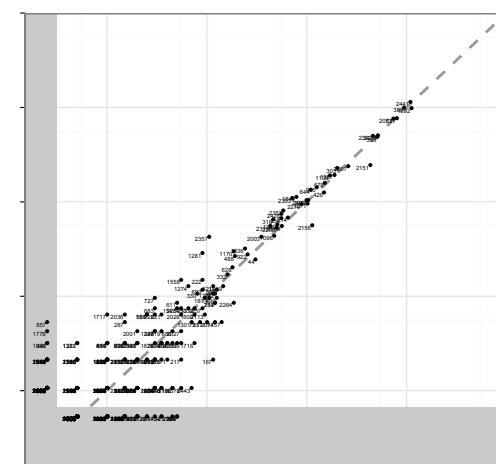

Extraction 2 vs. 3

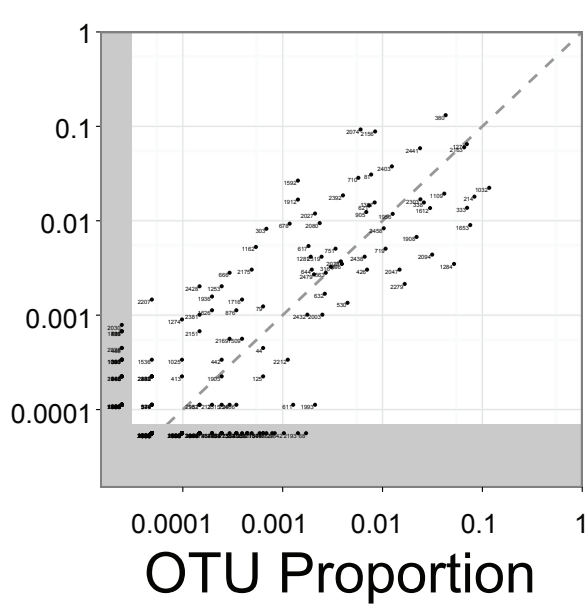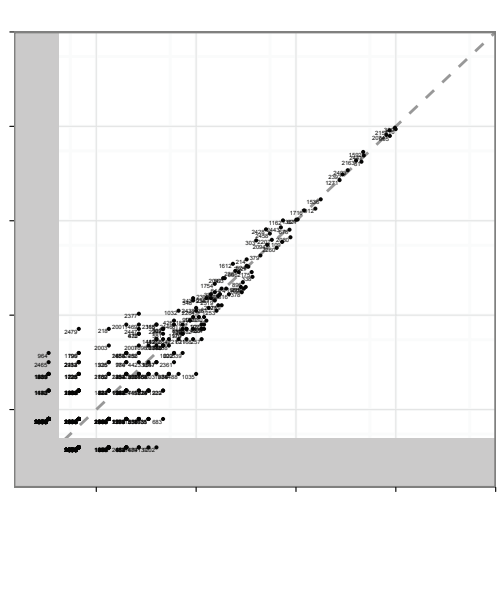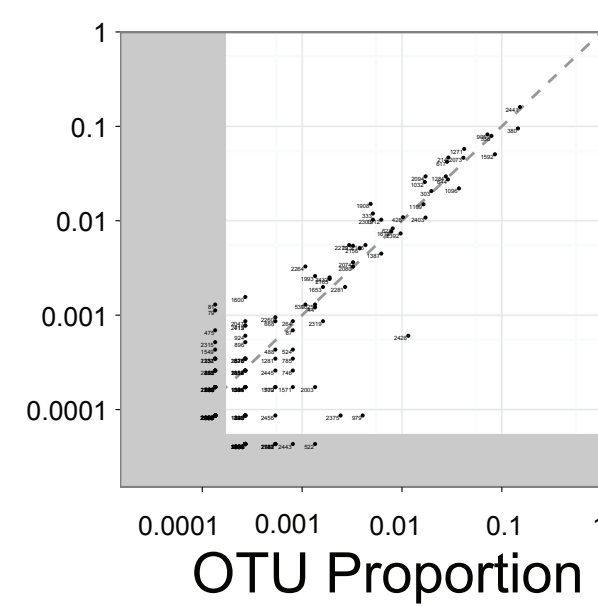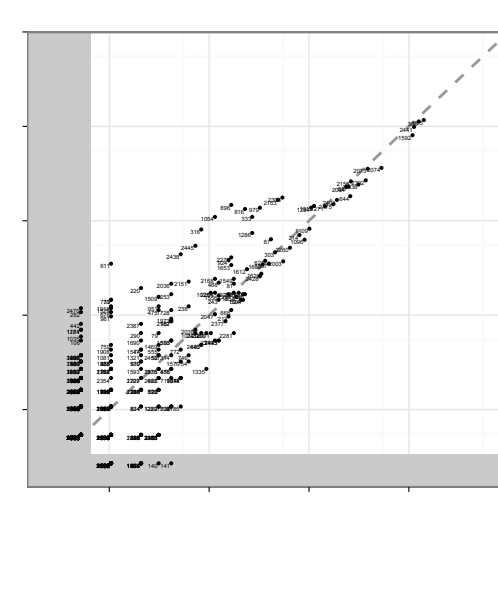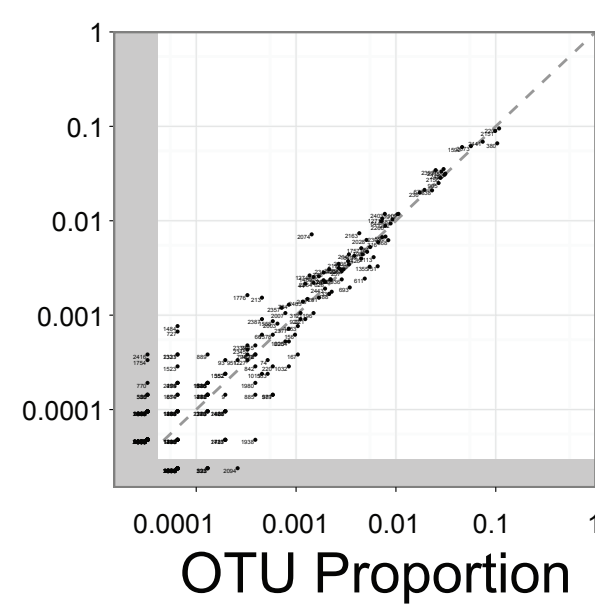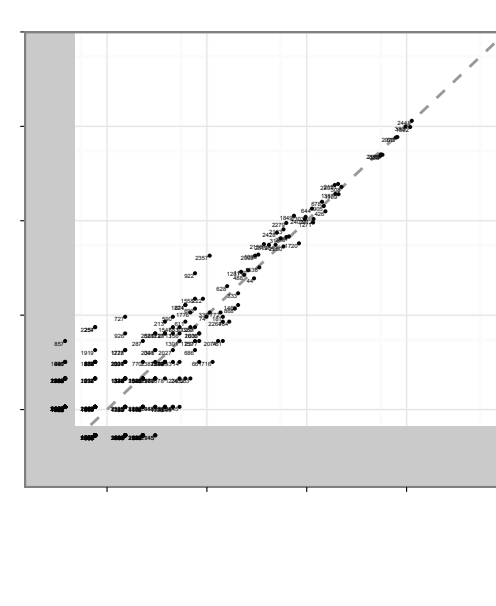

Supplement: Figure S1 — Complete set of bivariate plots demonstrating OTU reproducibility for each replicate and all subjects. (PDF) [file pone.0042786.s001.pdf]

OTU critical proportion for 95% reproducibility

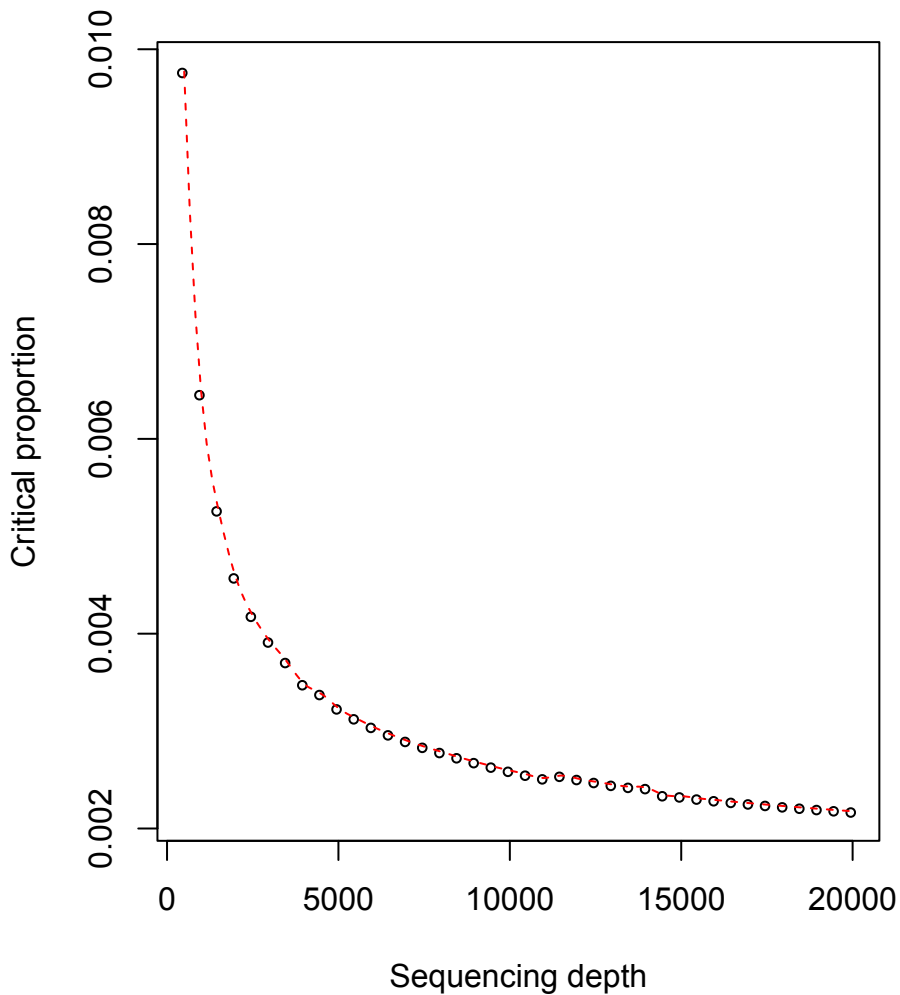

OTU critical counts for 95% reproducibility

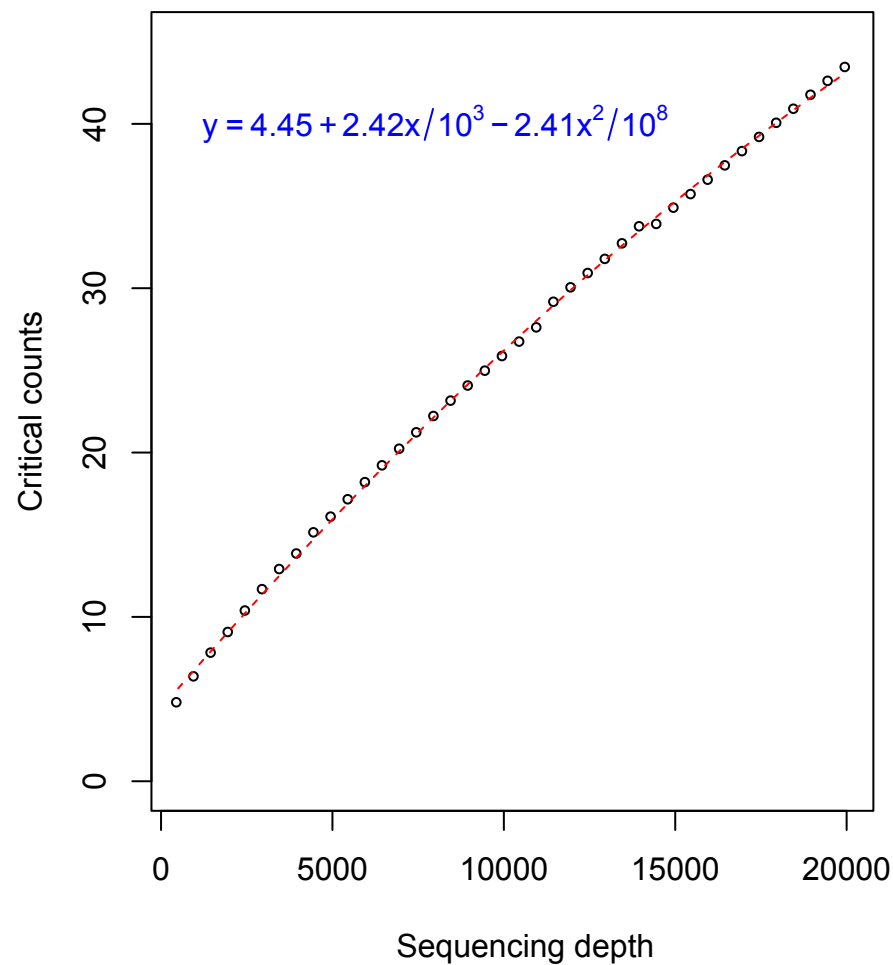

Supplement: Figure S2 — Fit of the empirical function to calculate the critical OTU count at various sequencing depths. (PDF) [file pone.0042786.s002.pdf]

Figure S3

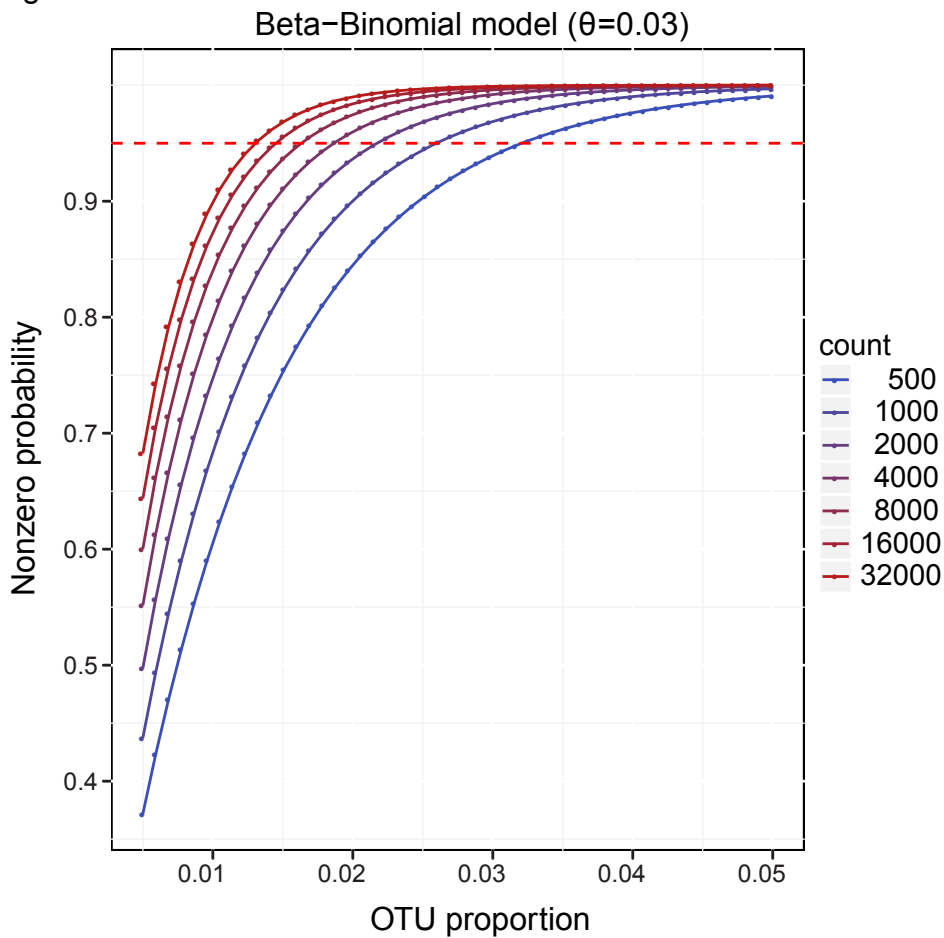

Supplement: Figure S3 — Non-zero probability of observing an OTU upon repeat sequencing 16S rDNA under the worst-case scenario. (PDF) [file pone.0042786.s003.pdf]

Figure S4

TX 20: BAL x OW

TX 26: BAL x OW

Healthy Subjects

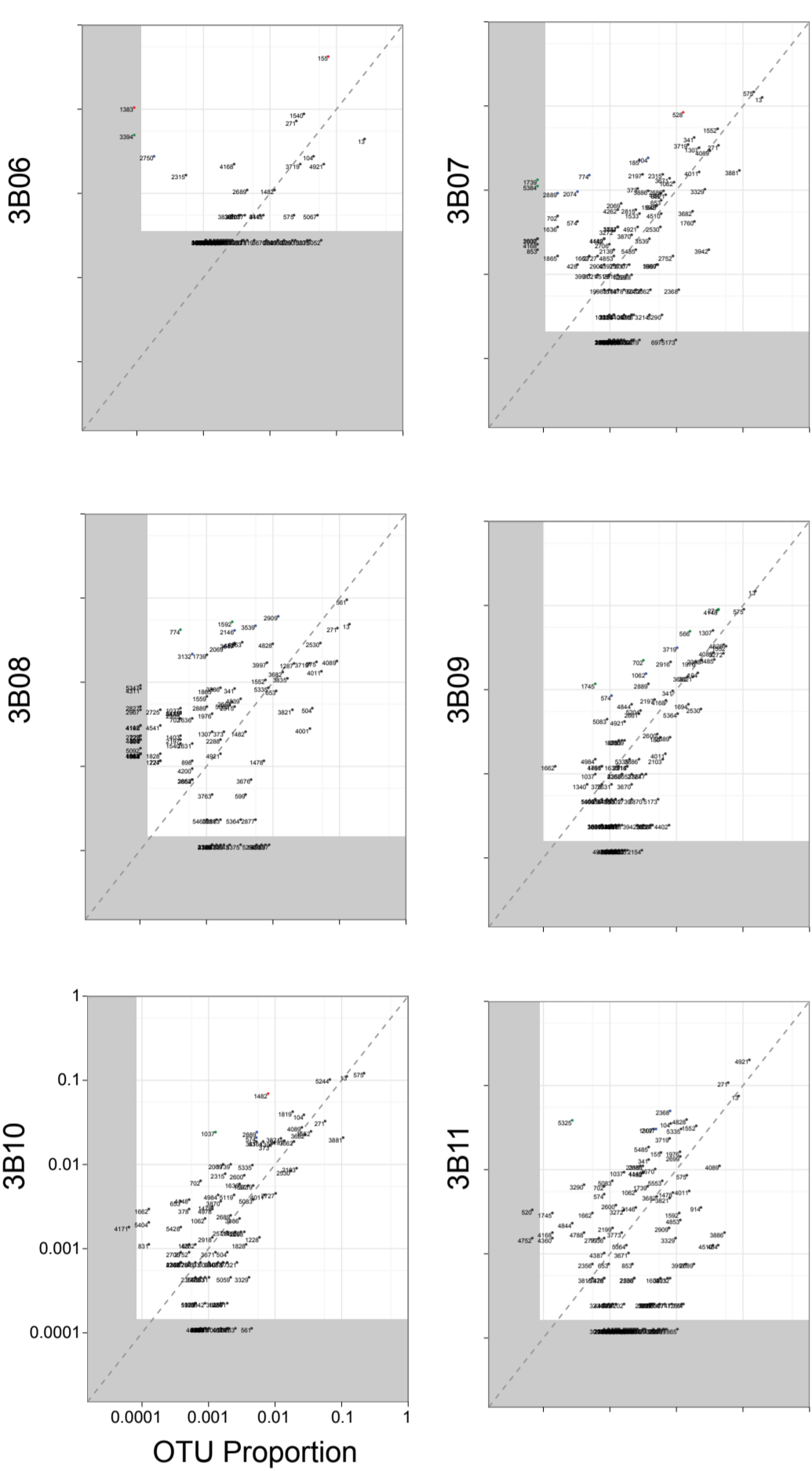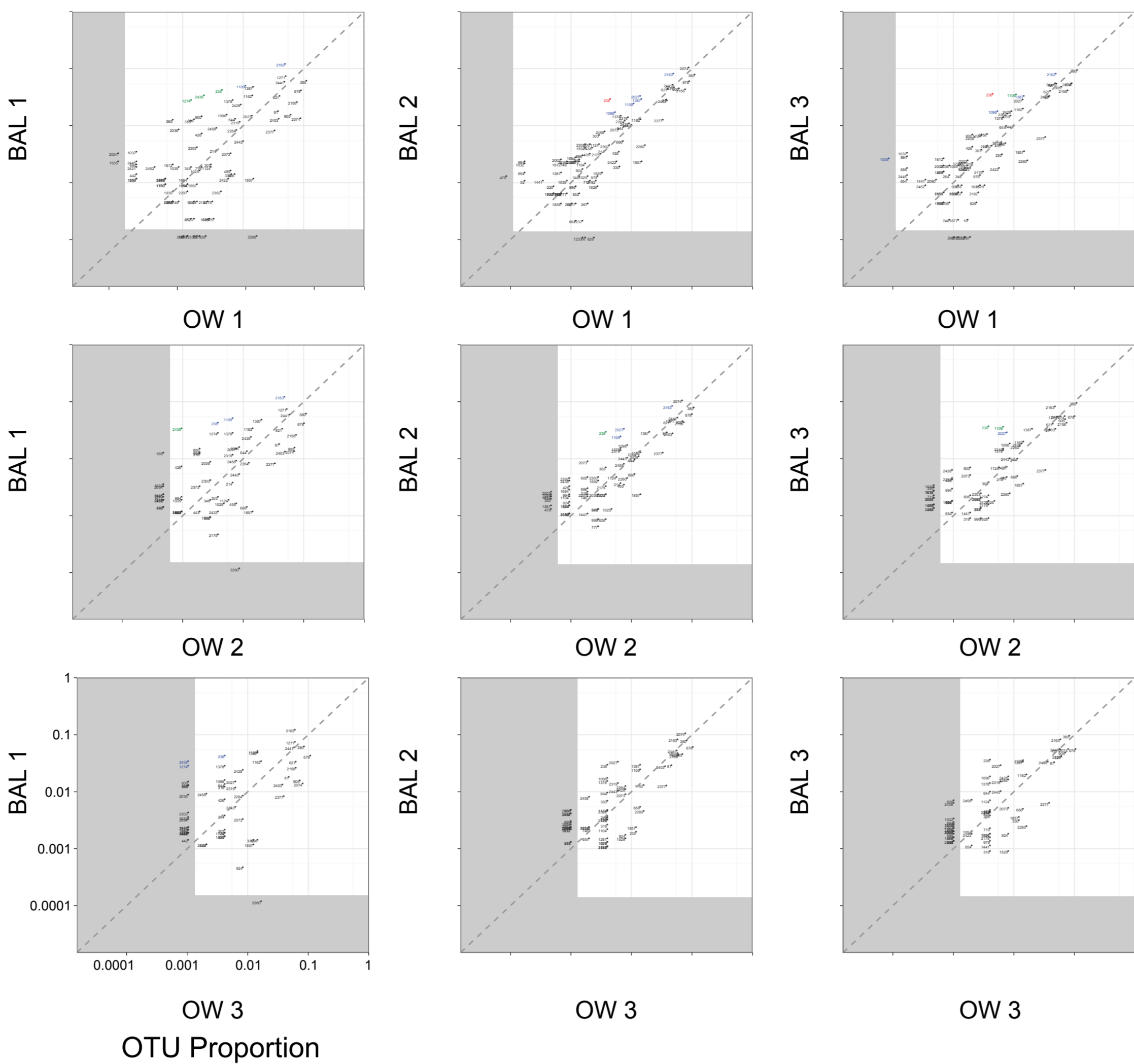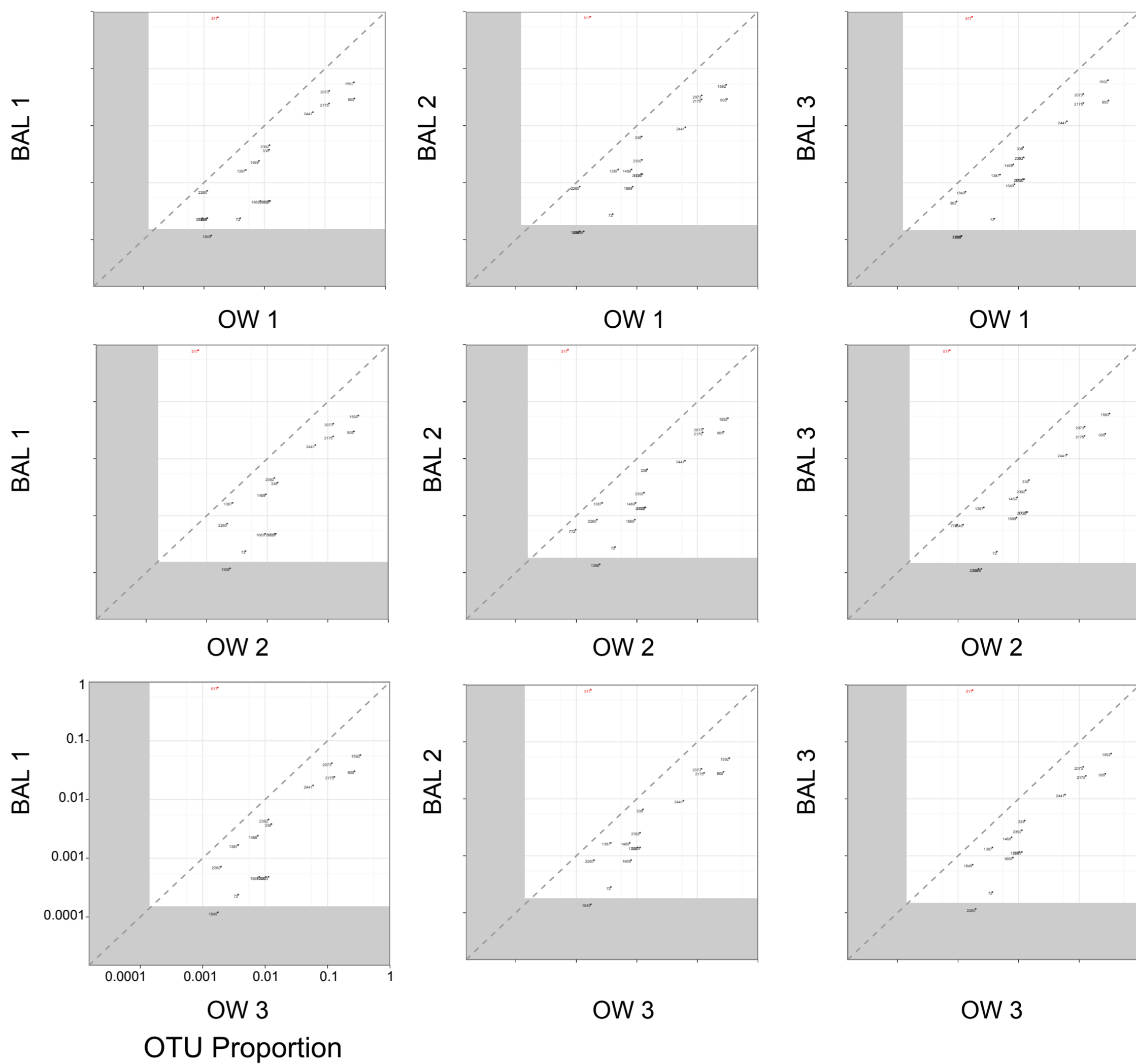

TX 43: BAL x OW

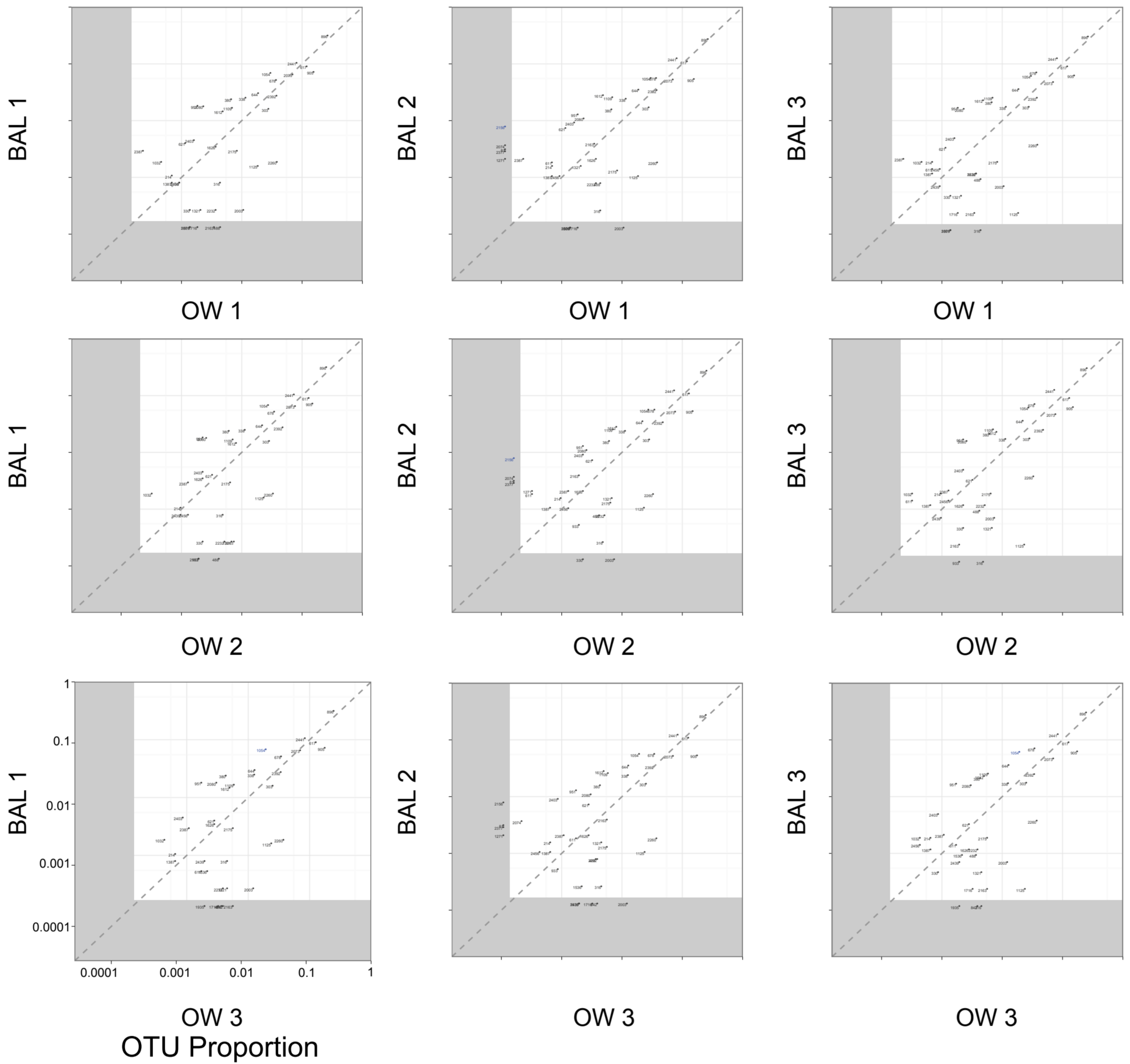

Pulm 1: BAL x OW

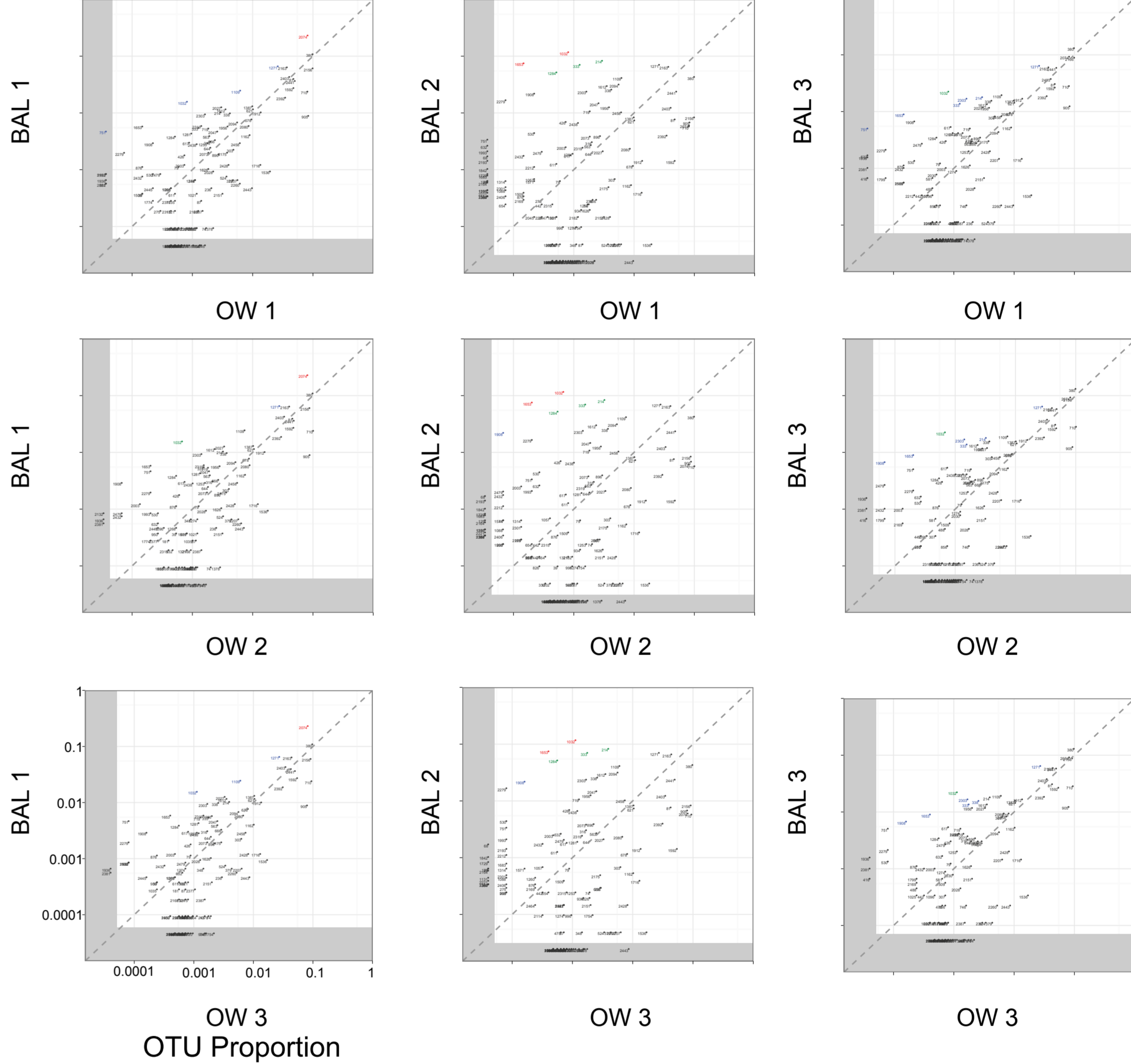

Pulm 3: BAL x OW

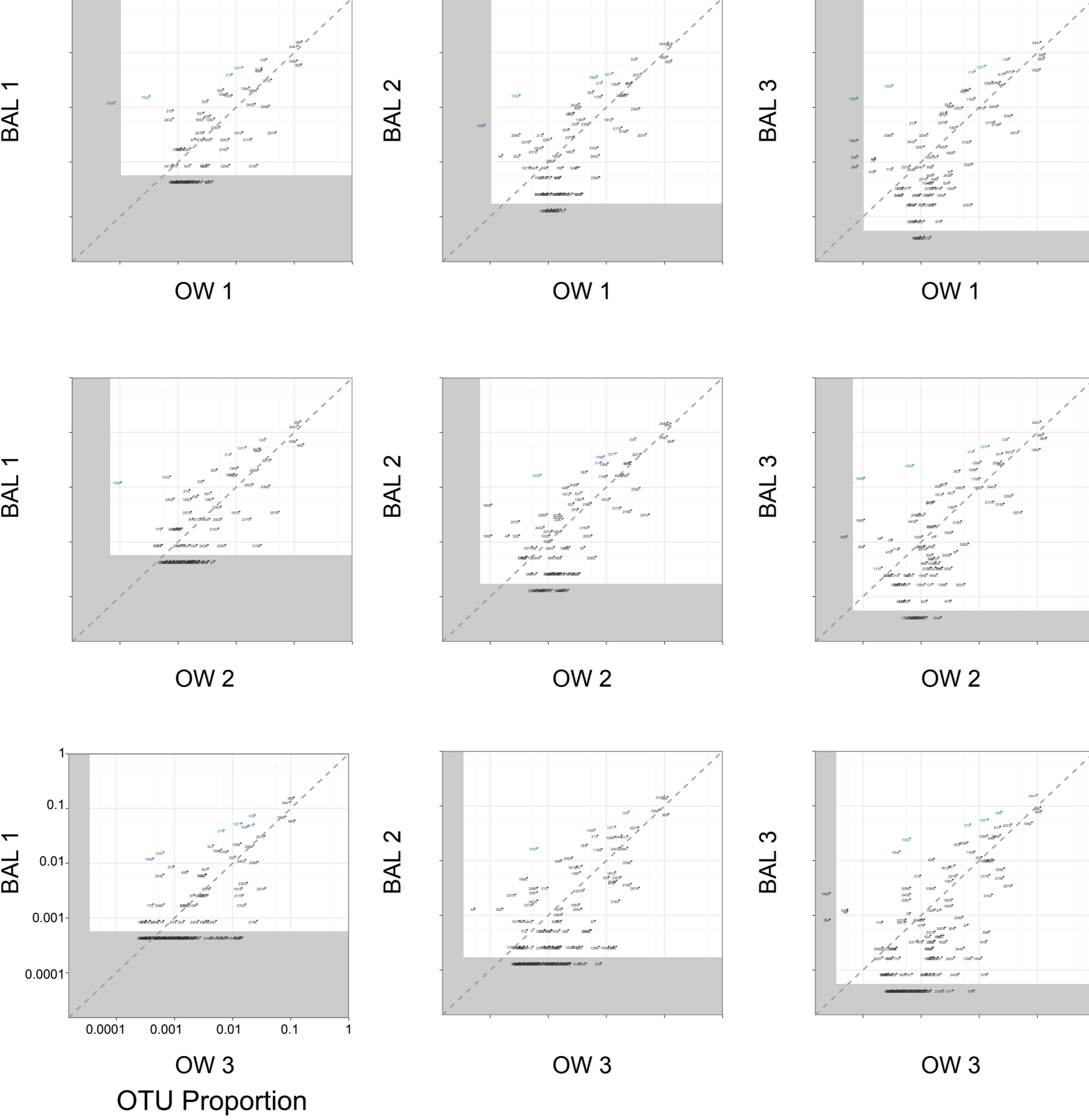

Pulm 4: BAL x OW

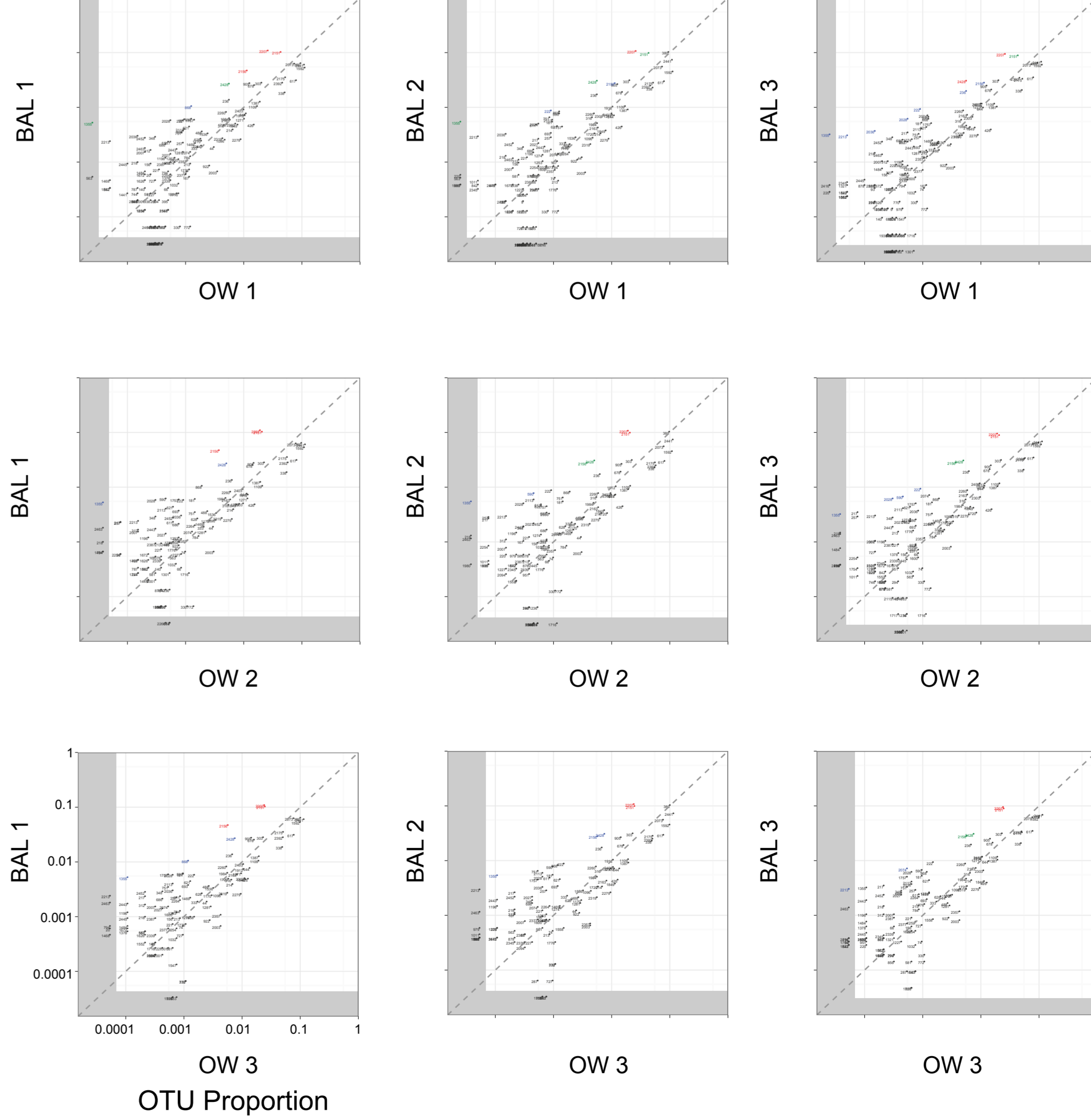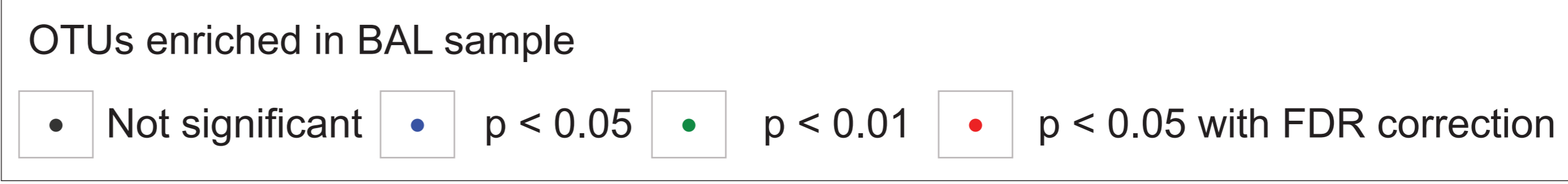

Supplement: Figure S4 — Complete set of outlier plots for pairwise BAL-OW comparisons. (PDF) [file pone.0042786.s004.pdf]

Figure S5

A. 3B08

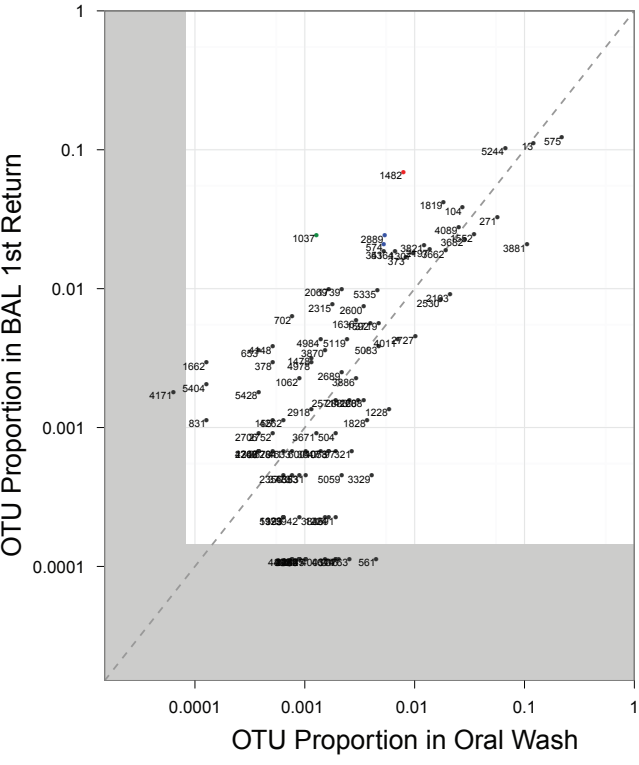

B. 3B09

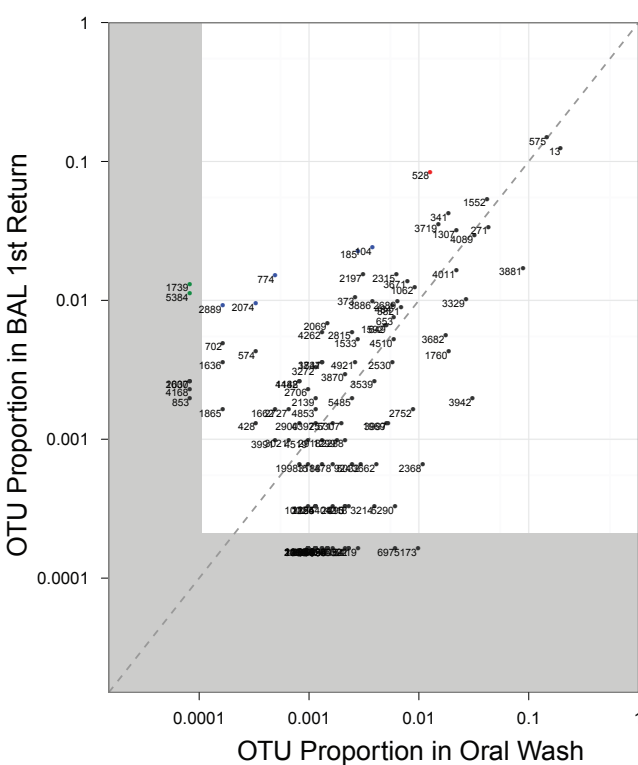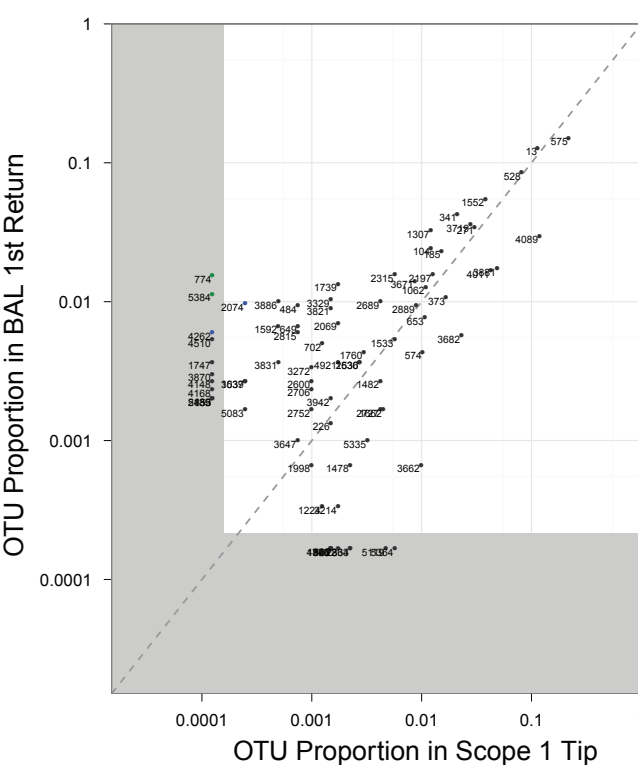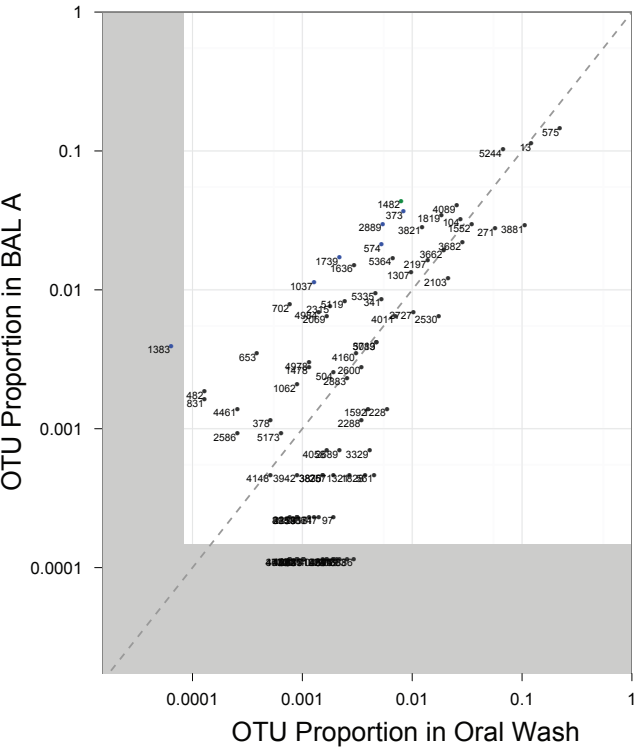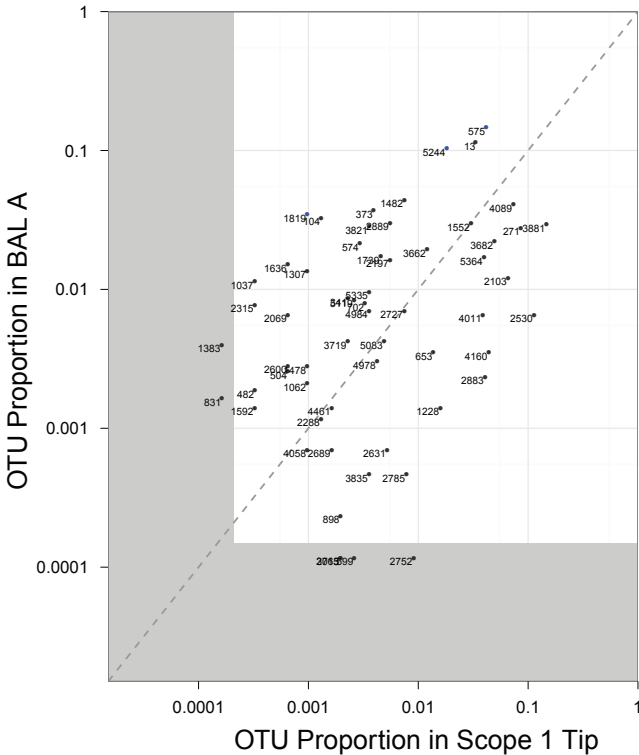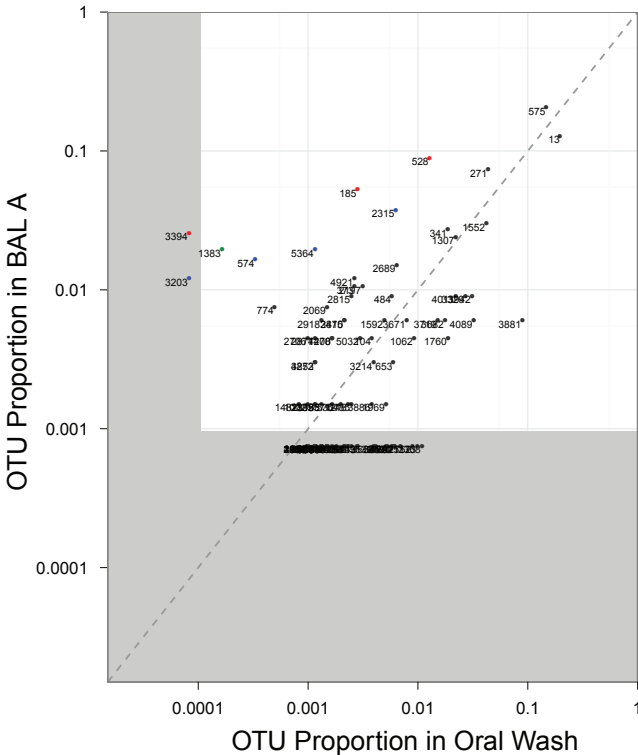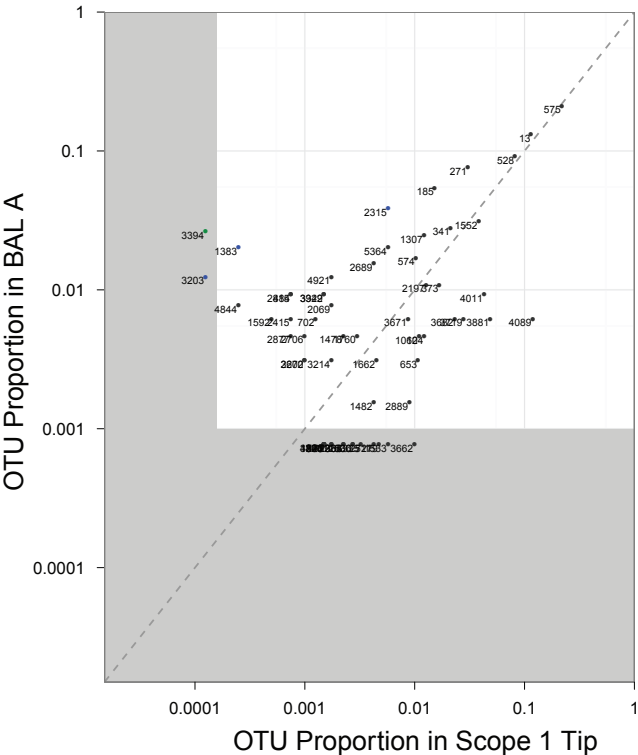

OTUs enriched in BAL sample

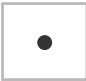

Not significant

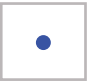

p < 0.05

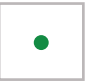

p < 0.01

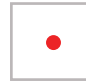

p < 0.05 with FDR correction

Supplement: Figure S5 — Healthy patient 3B08 and 3B09 outlier plots. (PDF) [file pone.0042786.s005.pdf]
