# Supplementary material for: Assessing Bacterial Populations in the Lung by Replicate Analysis of Samples from the Upper and Lower Respiratory Tracts
Source: PLoS One. 2012 Sep 6;7(9):e42786. doi: 10.1371/journal.pone.0042786 (PMC3435383; doi:10.1371/journal.pone.0042786)
Supplement: Table S1 — Summary of subjects and samples studied. (PDF) [file pone.0042786.s006.pdf]

Table S1

Sample ID	Clinical Status	Bacteria Cultured from BAL	Sample Type	# Reads
**Tx 20**	Lung transplant due to Sarcoid	*Staphylococcus aureus*	BAL1	4336
			BAL2	4693
			BAL3	4503
			OW1	5925
			OW2	1108
			OW3	516
**Tx 26**	Lung transplant due to Cystic Fibrosis	*Pseudomonas aeruginosa*	BAL1	4142
			BAL2	3523
			BAL3	4260
			OW1	5225
			OW2	4075
			OW3	4550
**Tx 43**	Lung transplant due to Chronic Obstructive Pulmonary Disease	None	BAL1	3735
			BAL2	3842
			BAL3	4241
			OW1	4373
			OW2	3158
			OW3	4652
**Pulm 1**	Sarcoid	None	BAL1	10604
			BAL2	20403
			BAL3	8890
			OW1	13656
			OW2	14992
			OW3	12574
**Pulm 3**	Pulmonary nodule / adenocarcinoma	None	BAL1	1131
			BAL2	3712
			BAL3	11575
			OW1	6309
			OW2	9614
			OW3	18560
**Pulm 4**	Bronchiolitis Obliterans Organizing Pneumonia	*Staphylococcus aureus*	BAL1	14913
			BAL2	15368
			BAL3	20895
			OW1	19909
			OW2	13148
			OW3	9372
