# Supplementary material for: Assessing Bacterial Populations in the Lung by Replicate Analysis of Samples from the Upper and Lower Respiratory Tracts
Source: PLoS One. 2012 Sep 6;7(9):e42786. doi: 10.1371/journal.pone.0042786 (PMC3435383; doi:10.1371/journal.pone.0042786)
Supplement: Table S2 — 16S rDNA OTU table. (PDF) [file pone.0042786.s007.pdf]

Table S2

QIIME v1.3.0-dev OTU table

#OTU ID	Consensus Lineage
0	Bacteria;Firmicutes;Clostridia;Clostridiales;Incertae Sedis XIV;Blautia
1	Bacteria;Bacteroidetes;Bacteroidia;Bacteroidales;Prevotellaceae;Prevotella
2	Bacteria;Proteobacteria;Betaproteobacteria;Burkholderiales;Alcaligenaceae;Alcaligenes
3	Bacteria;Actinobacteria;Actinobacteria;Actinomycetales
4	Bacteria;Acidobacteria;Acidobacteria_Gp6;Gp6
5	Bacteria;Fusobacteria;Fusobacteria;Fusobacteriales;Leptotrichiaceae;Leptotrichia
6	Bacteria;Firmicutes;Clostridia;Clostridiales;Veillonellaceae;Veillonella
7	Bacteria;Actinobacteria;Actinobacteria;Actinomycetales;Micrococcaceae;Arthrobacter
8	Bacteria;Fusobacteria;Fusobacteria;Fusobacteriales;Leptotrichiaceae
9	Bacteria;Actinobacteria;Actinobacteria;Actinomycetales;Corynebacteriaceae;Corynebacterium
10	Bacteria;Firmicutes;Bacilli;Lactobacillales
11	Bacteria;Firmicutes;Bacilli;Bacillales;Bacillaceae
12	Bacteria;Proteobacteria;Alphaproteobacteria;Sphingomonadales;Sphingomonadaceae;Sphingopyxis
13	Bacteria;Actinobacteria;Actinobacteria;Actinomycetales;Streptosporangiaceae;Microbispora
14	Bacteria;Firmicutes;Bacilli;Lactobacillales;Lactobacillaceae;Lactobacillus
15	Bacteria;Bacteroidetes;Bacteroidia;Bacteroidales;Prevotellaceae;Prevotella
16	Bacteria;Bacteroidetes;Flavobacteria;Flavobacteriales;Flavobacteriaceae;Capnocytophaga
17	Bacteria;Firmicutes;Clostridia;Clostridiales;Incertae Sedis XI;Parvimonas
18	Bacteria;Firmicutes;Bacilli;Lactobacillales;Carnobacteriaceae;Granulicatella
19	Bacteria;Firmicutes;Bacilli;Bacillales;Staphylococcaceae;Gemella
20	Bacteria;Bacteroidetes;Bacteroidia;Bacteroidales;Prevotellaceae;Prevotella
21	Bacteria
22	Bacteria;Bacteroidetes;Sphingobacteria;Sphingobacteriales;Sphingobacteriaceae;Mucilaginibacter
23	Bacteria;Bacteroidetes;Sphingobacteria;Sphingobacteriales;Cytophagaceae;Adhaeribacter
24	Bacteria;Firmicutes;Bacilli;Bacillales;Bacillaceae;Jeotgalibacillus
25	Bacteria;Actinobacteria;Actinobacteria;Coriobacteriales;Coriobacteriaceae;Atopobium
26	Bacteria;Proteobacteria;Gammaproteobacteria;Pseudomonadales;Moraxellaceae;Psychrobacter
27	Bacteria;Firmicutes;Clostridia;Clostridiales;Eubacteriaceae;Pseudoramibacter
28	Bacteria;Firmicutes;Clostridia;Clostridiales;Lachnospiraceae;Catonella
29	Bacteria;Proteobacteria;Alphaproteobacteria
30	Bacteria
31	Bacteria
32	Bacteria
33	Bacteria;Firmicutes;Bacilli;Bacillales
34	Bacteria
35	Bacteria;Firmicutes;Bacilli;Bacillales;Paenibacillaceae;Brevibacillus
36	Bacteria;Firmicutes
37	Bacteria;Actinobacteria;Actinobacteria;Actinomycetales;Nocardioidaceae;Marmoricola
38	Bacteria;Firmicutes;Clostridia;Clostridiales;Lachnospiraceae;Shuttleworthia
39	Bacteria;Fusobacteria;Fusobacteria;Fusobacteriales;Leptotrichiaceae;Leptotrichia
40	Bacteria;Firmicutes;Clostridia;Clostridiales;Lachnospiraceae;Oribacterium
41	Bacteria;Firmicutes;Clostridia;Clostridiales
42	Bacteria;Bacteroidetes;Flavobacteria;Flavobacteriales;Flavobacteriaceae
43	Bacteria;Actinobacteria;Actinobacteria
44	Bacteria;Firmicutes;Clostridia;Clostridiales;Incertae Sedis XIII;Mogibacterium
45	Bacteria;Actinobacteria;Actinobacteria;Actinomycetales
46	Bacteria;Bacteroidetes;Flavobacteria;Flavobacteriales;Flavobacteriaceae
47	Bacteria
48	Bacteria;Bacteroidetes
49	Bacteria;Bacteroidetes;Bacteroidia;Bacteroidales
50	Bacteria;Firmicutes;Bacilli;Bacillales;Paenibacillaceae;Paenibacillus
51	Bacteria;Firmicutes;Clostridia;Clostridiales;Veillonellaceae;Veillonella
52	Bacteria;Firmicutes;Clostridia;Clostridiales;Veillonellaceae;Megasphaera
53	Bacteria;Proteobacteria;Gammaproteobacteria;Alteromonadales;Alteromonadaceae;Alishewanella
54	Bacteria;Firmicutes;Clostridia;Clostridiales;Lachnospiraceae;Oribacterium
55	Bacteria
56	Bacteria
57	Bacteria
58	Bacteria;Firmicutes;Clostridia;Clostridiales;Incertae Sedis XIII;Mogibacterium
59	Bacteria;Proteobacteria;Alphaproteobacteria;Rhizobiales;Beijerinckiaceae
60	Bacteria
61	Bacteria;Proteobacteria;Betaproteobacteria;Neisseriales;Neisseriaceae;Neisseria
62	Bacteria;Actinobacteria;Actinobacteria;Actinomycetales;Pseudonocardiaceae;Saccharomonospora
63	Bacteria;Firmicutes;Bacilli;Lactobacillales;Aerococcaceae;Abiotrophia
64	Bacteria;Bacteroidetes;Flavobacteria;Flavobacteriales;Flavobacteriaceae;Capnocytophaga
65	Bacteria;Firmicutes;Clostridia;Clostridiales;Veillonellaceae;Megasphaera
66	Bacteria;Bacteroidetes;Bacteroidia;Bacteroidales;Porphyromonadaceae;Porphyromonas
67	Bacteria;Bacteroidetes;Bacteroidia;Bacteroidales;Prevotellaceae
68	Bacteria;Firmicutes;Clostridia;Clostridiales;Veillonellaceae;Selenomonas
69	Bacteria;Bacteroidetes;Bacteroidia;Bacteroidales;Prevotellaceae;Prevotella
70	Bacteria;Bacteroidetes;Bacteroidia;Bacteroidales;Porphyromonadaceae;Porphyromonas
71	Bacteria;Proteobacteria;Betaproteobacteria;Hydrogenophilales;Hydrogenophilaceae;Petrobacter
72	Bacteria;Actinobacteria;Actinobacteria;Actinomycetales;Actinomycetaceae;Actinomyces
73	Bacteria;Actinobacteria;Actinobacteria;Actinomycetales;Mycobacteriaceae;Mycobacterium
74	Bacteria;Bacteroidetes;Bacteroidia;Bacteroidales;Prevotellaceae;Prevotella
75	Bacteria;Bacteroidetes;Bacteroidia;Bacteroidales;Porphyromonadaceae
76	Bacteria;Proteobacteria;Betaproteobacteria;Burkholderiales;Comamonadaceae;Schlegelella
77	Bacteria;Proteobacteria;Betaproteobacteria;Burkholderiales;Oxalobacteraceae
78	Bacteria;Firmicutes;Bacilli;Lactobacillales;Lactobacillaceae;Lactobacillus
79	Bacteria;TM7;TM7_genera_incertae_sedis
80	Bacteria;Actinobacteria;Actinobacteria;Actinomycetales;Micrococcaceae;Kocuria
81	Bacteria;Bacteroidetes;Bacteroidia;Bacteroidales
82	Bacteria;Firmicutes
83	Bacteria;Firmicutes;Clostridia;Clostridiales;Lachnospiraceae
84	Bacteria
85	Bacteria
86	Bacteria;Bacteroidetes;Bacteroidia;Bacteroidales;Prevotellaceae;Prevotella
87	Bacteria;Proteobacteria;Gammaproteobacteria;Pasteurellales;Pasteurellaceae;Aggregatibacter
88	Bacteria;Firmicutes;Clostridia;Clostridiales;Lachnospiraceae;Moryella
89	Bacteria;Actinobacteria;Actinobacteria;Actinomycetales;Actinomycetaceae;Actinomyces
90	Bacteria;Fusobacteria;Fusobacteria;Fusobacteriales;Fusobacteriaceae;Fusobacterium
91	Bacteria
92	Bacteria
93	Bacteria;Bacteroidetes
94	Bacteria;Actinobacteria;Actinobacteria;Actinomycetales;Micrococcaceae;Kocuria
95	Unclassified
96	Bacteria;Actinobacteria;Actinobacteria;Actinomycetales;Nocardiaceae;Rhodococcus
97	Bacteria;SR1;SR1_genera_incertae_sedis
98	Bacteria;Firmicutes;Bacilli;Lactobacillales;Streptococcaceae;Streptococcus
99	Bacteria;Proteobacteria;Alphaproteobacteria;Rhizobiales;Aurantimonadaceae;Aurantimonas
100	Bacteria
101	Bacteria;Firmicutes;Bacilli;Bacillales;Paenibacillaceae
102	Bacteria;Bacteroidetes;Bacteroidia;Bacteroidales;Prevotellaceae;Prevotella
103	Bacteria;Bacteroidetes;Flavobacteria;Flavobacteriales;Flavobacteriaceae;Chryseobacterium
104	Bacteria;Firmicutes;Bacilli;Bacillales;Staphylococcaceae;Gemella
105	Bacteria;Bacteroidetes
106	Bacteria;Actinobacteria;Actinobacteria;Actinomycetales;Actinomycetaceae;Actinomyces
107	Bacteria;Firmicutes;Bacilli;Bacillales;Bacillaceae;Bacillus
108	Bacteria;Proteobacteria;Betaproteobacteria;Neisseriales;Neisseriaceae;Neisseria
109	Bacteria
110	Bacteria;Bacteroidetes
111	Bacteria;Actinobacteria;Actinobacteria;Actinomycetales
112	Bacteria;Bacteroidetes;Bacteroidia;Bacteroidales;Porphyromonadaceae;Porphyromonas
113	Bacteria;Bacteroidetes;Bacteroidia;Bacteroidales;Prevotellaceae;Prevotella
114	Bacteria;Firmicutes;Clostridia;Clostridiales;Lachnospiraceae;Moryella
115	Bacteria;Proteobacteria;Alphaproteobacteria;Rhodospirillales;Acetobacteraceae
116	Bacteria;Proteobacteria;Betaproteobacteria;Burkholderiales;Oxalobacteraceae;Massilia
117	Bacteria;Actinobacteria;Actinobacteria;Actinomycetales;Microbacteriaceae;Microbacterium
118	Bacteria;Bacteroidetes;Sphingobacteria;Sphingobacteriales;Chitinophagaceae;Flavisolibacter
119	Bacteria;Proteobacteria;Alphaproteobacteria;Rhizobiales;Methylobacteriaceae;Methylobacterium
120	Bacteria;Firmicutes;Clostridia;Clostridiales;Veillonellaceae;Veillonella
121	Bacteria;Firmicutes;Bacilli;Lactobacillales;Streptococcaceae;Streptococcus
122	Bacteria;Bacteroidetes;Bacteroidia;Bacteroidales;Prevotellaceae;Prevotella
123	Bacteria;Actinobacteria;Actinobacteria;Actinomycetales
124	Bacteria;TM7;TM7_genera_incertae_sedis
125	Bacteria;Firmicutes;Clostridia;Clostridiales
126	Bacteria;Proteobacteria;Deltaproteobacteria;Myxococcales;Polyangiaceae;Sorangium
127	Bacteria;Verrucomicrobia;Subdivision3;Subdivision3_genera_incertae_sedis
128	Bacteria;Firmicutes;Bacilli;Lactobacillales;Streptococcaceae;Streptococcus
129	Bacteria;Proteobacteria;Gammaproteobacteria;Enterobacteriales;Enterobacteriaceae
130	Bacteria;Firmicutes;Bacilli;Lactobacillales;Carnobacteriaceae;Granulicatella
131	Bacteria;Bacteroidetes;Sphingobacteria;Sphingobacteriales;Chitinophagaceae
132	Bacteria;Proteobacteria;Alphaproteobacteria;Rhodobacterales;Rhodobacteraceae;Paracoccus
133	Bacteria
134	Bacteria
135	Bacteria;Bacteroidetes
136	Bacteria
137	Bacteria
138	Bacteria;Firmicutes;Bacilli;Bacillales;Paenibacillaceae;Brevibacillus
139	Bacteria;Proteobacteria;Betaproteobacteria;Burkholderiales
140	Bacteria;Actinobacteria;Actinobacteria;Actinomycetales;Micrococcaceae;Rothia
141	Bacteria;Fusobacteria;Fusobacteria;Fusobacteriales;Fusobacteriaceae;Fusobacterium
142	Bacteria;Actinobacteria;Actinobacteria;Acidimicrobiales;Iamiaceae;Iamia
143	Bacteria;Proteobacteria;Betaproteobacteria;Burkholderiales;Comamonadaceae;Pelomonas
144	Bacteria;Firmicutes;Bacilli;Lactobacillales;Streptococcaceae;Streptococcus
145	Bacteria;Bacteroidetes
146	Bacteria;Bacteroidetes;Sphingobacteria;Sphingobacteriales;Chitinophagaceae
147	Bacteria;Firmicutes;Bacilli;Lactobacillales;Carnobacteriaceae;Alloiococcus
148	Bacteria;Actinobacteria;Actinobacteria;Actinomycetales
149	Bacteria;Firmicutes;Bacilli;Lactobacillales;Streptococcaceae;Streptococcus
150	Bacteria;Actinobacteria;Actinobacteria;Actinomycetales
151	Bacteria;Firmicutes;Clostridia;Clostridiales;Veillonellaceae;Dialister
152	Bacteria;Firmicutes;Clostridia;Clostridiales;Veillonellaceae
153	Bacteria;Fusobacteria;Fusobacteria;Fusobacteriales;Fusobacteriaceae;Fusobacterium
154	Bacteria;Bacteroidetes;Flavobacteria;Flavobacteriales;Flavobacteriaceae
155	Bacteria
156	Bacteria;Firmicutes;Clostridia;Clostridiales;Incertae Sedis XI;Parvimonas
157	Bacteria;Fusobacteria;Fusobacteria;Fusobacteriales;Leptotrichiaceae
158	Bacteria;Bacteroidetes;Flavobacteria;Flavobacteriales;Flavobacteriaceae;Capnocytophaga
159	Bacteria;Proteobacteria;Betaproteobacteria
160	Bacteria;Actinobacteria;Actinobacteria;Coriobacteriales;Coriobacteriaceae;Atopobium
161	Bacteria;Firmicutes;Bacilli;Lactobacillales;Leuconostocaceae;Weissella
162	Bacteria;Actinobacteria;Actinobacteria;Actinomycetales;Pseudonocardiaceae
163	Bacteria;Actinobacteria;Actinobacteria;Actinomycetales;Micrococcaceae;Rothia
164	Bacteria;Firmicutes;Bacilli;Bacillales;Bacillaceae
165	Bacteria;Actinobacteria;Actinobacteria;Actinomycetales;Geodermatophilaceae;Modestobacter
166	Bacteria;Bacteroidetes;Bacteroidia;Bacteroidales;Prevotellaceae;Prevotella
167	Bacteria;Bacteroidetes;Bacteroidia;Bacteroidales;Prevotellaceae;Prevotella
168	Bacteria;Firmicutes;Bacilli;Lactobacillales;Streptococcaceae;Streptococcus
169	Bacteria;Firmicutes;Bacilli;Bacillales;Bacillaceae;Bacillus
170	Bacteria
171	Bacteria
172	Bacteria;Actinobacteria;Actinobacteria;Actinomycetales;Propionibacteriaceae;Microlunatus
173	Bacteria;Actinobacteria;Actinobacteria;Actinomycetales;Streptomycetaceae;Streptomyces
174	Bacteria;Firmicutes;Clostridia;Clostridiales;Veillonellaceae;Veillonella
175	Bacteria;Proteobacteria;Alphaproteobacteria;Sphingomonadales;Sphingomonadaceae
176	Bacteria;Actinobacteria;Actinobacteria
177	Bacteria;Proteobacteria;Alphaproteobacteria;Rhizobiales;Hyphomicrobiaceae;Hyphomicrobium
178	Bacteria;Firmicutes;Clostridia;Clostridiales;Veillonellaceae;Veillonella
179	Bacteria;Bacteroidetes;Bacteroidia;Bacteroidales;Prevotellaceae;Prevotella
180	Bacteria;Proteobacteria;Betaproteobacteria;Burkholderiales;Alcaligenaceae;Bordetella
181	Bacteria;Firmicutes;Clostridia;Clostridiales
182	Bacteria;Actinobacteria;Actinobacteria;Solirubrobacterales;Solirubrobacteraceae;Solirubrobacter
183	Bacteria;Proteobacteria;Betaproteobacteria
184	Bacteria;Firmicutes
185	Bacteria;Bacteroidetes;Bacteroidia;Bacteroidales;Prevotellaceae;Prevotella
186	Bacteria;Firmicutes;Clostridia;Clostridiales;Veillonellaceae
187	Bacteria;Bacteroidetes;Bacteroidia;Bacteroidales;Prevotellaceae;Prevotella
188	Bacteria;Proteobacteria;Gammaproteobacteria;Pseudomonadales;Moraxellaceae;Alkanindiges
189	Bacteria;Proteobacteria;Gammaproteobacteria
190	Bacteria;Fusobacteria;Fusobacteria;Fusobacteriales
191	Bacteria;Proteobacteria;Alphaproteobacteria;Rhizobiales;Methylobacteriaceae;Methylobacterium
192	Bacteria;Firmicutes;Bacilli;Bacillales;Thermoactinomycetaceae;Thermoactinomyces
193	Bacteria;Bacteroidetes;Bacteroidia;Bacteroidales;Prevotellaceae;Prevotella
194	Bacteria;Actinobacteria;Actinobacteria;Actinomycetales
195	Bacteria;Actinobacteria;Actinobacteria;Solirubrobacterales
196	Bacteria;Firmicutes;Bacilli;Lactobacillales;Streptococcaceae;Streptococcus
197	Bacteria
198	Bacteria;Firmicutes;Bacilli;Bacillales;Staphylococcaceae
199	Bacteria;Firmicutes;Bacilli;Lactobacillales;Streptococcaceae;Streptococcus
200	Bacteria;Firmicutes;Bacilli;Bacillales;Planococcaceae;Kurthia
201	Bacteria;Firmicutes;Bacilli;Lactobacillales;Aerococcaceae;Aerococcus
202	Bacteria;Bacteroidetes;Bacteroidia;Bacteroidales
203	Bacteria;Bacteroidetes;Bacteroidia;Bacteroidales;Prevotellaceae
204	Bacteria;Firmicutes;Bacilli;Lactobacillales;Streptococcaceae;Streptococcus
205	Bacteria;Firmicutes;Clostridia;Clostridiales;Veillonellaceae;Veillonella
206	Bacteria;Actinobacteria;Actinobacteria;Actinomycetales;Nocardioidaceae
207	Bacteria;Proteobacteria;Alphaproteobacteria
208	Bacteria
209	Bacteria;Bacteroidetes;Bacteroidia;Bacteroidales;Prevotellaceae;Prevotella
210	Bacteria;Firmicutes;Clostridia;Clostridiales;Incertae Sedis XI
211	Bacteria
212	Bacteria;Firmicutes;Clostridia;Clostridiales;Lachnospiraceae;Dorea
213	Bacteria;Bacteroidetes;Bacteroidia;Bacteroidales;Prevotellaceae;Prevotella
214	Bacteria;Bacteroidetes;Bacteroidia;Bacteroidales;Prevotellaceae;Prevotella
215	Bacteria;Firmicutes;Clostridia;Clostridiales;Lachnospiraceae
216	Bacteria;Firmicutes;Bacilli;Bacillales;Staphylococcaceae;Staphylococcus
217	Bacteria;Bacteroidetes;Bacteroidia;Bacteroidales;Bacteroidales_incertae_sedis;Phocaeicola
218	Bacteria;Firmicutes;Clostridia;Clostridiales;Eubacteriaceae;Eubacterium
219	Bacteria;Bacteroidetes;Bacteroidia;Bacteroidales;Prevotellaceae
220	Bacteria;TM7;TM7_genera_incertae_sedis
221	Bacteria;Bacteroidetes;Flavobacteria;Flavobacteriales;Flavobacteriaceae;Capnocytophaga
222	Bacteria;Proteobacteria;Gammaproteobacteria;Pasteurellales;Pasteurellaceae;Haemophilus
223	Bacteria
224	Bacteria;Firmicutes;Bacilli;Bacillales
225	Bacteria;Bacteroidetes;Sphingobacteria;Sphingobacteriales;Saprospiraceae
226	Bacteria
227	Bacteria;Actinobacteria;Actinobacteria;Actinomycetales;Microbacteriaceae;Microbacterium
228	Bacteria;Fusobacteria;Fusobacteria;Fusobacteriales;Fusobacteriaceae;Fusobacterium
229	Bacteria;Actinobacteria;Actinobacteria;Actinomycetales;Microbacteriaceae;Microbacterium
230	Bacteria;Firmicutes;Clostridia;Clostridiales;Peptostreptococcaceae;Peptostreptococcus
231	Bacteria;Bacteroidetes
232	Bacteria
233	Bacteria;Firmicutes;Bacilli;Lactobacillales;Streptococcaceae;Streptococcus
234	Bacteria;Firmicutes;Bacilli;Lactobacillales;Streptococcaceae;Streptococcus
235	Bacteria
236	Bacteria;Fusobacteria;Fusobacteria;Fusobacteriales;Fusobacteriaceae;Fusobacterium
237	Bacteria;Proteobacteria;Epsilonproteobacteria;Campylobacterales;Campylobacteraceae;Campylobacter
238	Bacteria;Firmicutes;Bacilli;Lactobacillales;Streptococcaceae;Streptococcus
239	Bacteria;Actinobacteria;Actinobacteria;Actinomycetales
240	Bacteria;Firmicutes;Clostridia;Clostridiales;Veillonellaceae;Dialister
241	Bacteria;Bacteroidetes;Bacteroidia;Bacteroidales;Prevotellaceae;Prevotella
242	Bacteria;Bacteroidetes;Sphingobacteria;Sphingobacteriales;Cytophagaceae;Dyadobacter
243	Bacteria;Proteobacteria;Gammaproteobacteria;Pasteurellales;Pasteurellaceae;Actinobacillus
244	Bacteria;Proteobacteria;Betaproteobacteria;Burkholderiales;Oxalobacteraceae;Duganella
245	Bacteria;Proteobacteria;Alphaproteobacteria;Rhizobiales;Methylobacteriaceae;Methylobacterium
246	Bacteria;Actinobacteria;Actinobacteria;Actinomycetales;Micrococcaceae;Rothia
247	Bacteria;Firmicutes;Clostridia;Clostridiales;Veillonellaceae
248	Bacteria;Firmicutes;Bacilli;Lactobacillales;Lactobacillaceae;Lactobacillus
249	Bacteria;Acidobacteria;Acidobacteria_Gp15;Gp15
250	Bacteria;Bacteroidetes;Bacteroidia;Bacteroidales;Prevotellaceae;Prevotella
251	Bacteria
252	Bacteria;TM7;TM7_genera_incertae_sedis
253	Bacteria;Proteobacteria;Alphaproteobacteria;Rhodobacterales;Rhodobacteraceae
254	Bacteria;Firmicutes;Clostridia;Clostridiales;Incertae Sedis XI;Anaerococcus
255	Bacteria;Firmicutes;Clostridia;Clostridiales;Veillonellaceae;Megasphaera
256	Bacteria;Firmicutes;Bacilli;Lactobacillales;Streptococcaceae;Streptococcus
257	Bacteria;Actinobacteria;Actinobacteria;Coriobacteriales;Coriobacteriaceae;Atopobium
258	Bacteria;Bacteroidetes;Bacteroidia;Bacteroidales
259	Bacteria;Bacteroidetes;Bacteroidia;Bacteroidales;Prevotellaceae;Prevotella
260	Bacteria;Bacteroidetes;Bacteroidia;Bacteroidales;Prevotellaceae;Prevotella
261	Bacteria
262	Bacteria;Actinobacteria;Actinobacteria;Actinomycetales;Brevibacteriaceae;Brevibacterium
263	Bacteria;Actinobacteria;Actinobacteria;Actinomycetales
264	Bacteria;Firmicutes;Clostridia;Clostridiales;Veillonellaceae;Dialister
265	Bacteria
266	Bacteria;Proteobacteria
267	Bacteria;TM7;TM7_genera_incertae_sedis
268	Bacteria;Actinobacteria;Actinobacteria;Actinomycetales
269	Bacteria;Firmicutes;Bacilli;Lactobacillales
270	Bacteria;TM7;TM7_genera_incertae_sedis
271	Bacteria;Proteobacteria;Alphaproteobacteria;Rhodospirillales;Rhodospirillaceae;Skermanella
272	Bacteria
273	Bacteria;Actinobacteria;Actinobacteria;Actinomycetales;Micromonosporaceae;Micromonospora
274	Bacteria;Actinobacteria;Actinobacteria;Actinomycetales;Microbacteriaceae;Leifsonia
275	Bacteria;Fusobacteria;Fusobacteria;Fusobacteriales;Fusobacteriaceae;Fusobacterium
276	Bacteria;Actinobacteria;Actinobacteria;Actinomycetales;Micrococcaceae;Arthrobacter
277	Bacteria;Firmicutes;Bacilli;Lactobacillales;Streptococcaceae;Streptococcus
278	Bacteria;Bacteroidetes;Bacteroidia;Bacteroidales;Prevotellaceae;Prevotella
279	Bacteria;Bacteroidetes;Bacteroidia;Bacteroidales;Porphyromonadaceae;Porphyromonas
280	Bacteria
281	Bacteria;Proteobacteria;Gammaproteobacteria
282	Bacteria;Bacteroidetes;Flavobacteria;Flavobacteriales;Flavobacteriaceae
283	Bacteria;Bacteroidetes;Bacteroidia;Bacteroidales;Porphyromonadaceae;Porphyromonas
284	Bacteria;TM7;TM7_genera_incertae_sedis
285	Bacteria;Actinobacteria;Actinobacteria;Actinomycetales;Micrococcaceae;Arthrobacter
286	Bacteria;Bacteroidetes;Bacteroidia;Bacteroidales;Prevotellaceae;Prevotella
287	Bacteria;Firmicutes;Bacilli;Lactobacillales;Streptococcaceae;Streptococcus
288	Bacteria;Firmicutes;Bacilli;Bacillales;Staphylococcaceae;Staphylococcus
289	Bacteria;Bacteroidetes;Bacteroidia;Bacteroidales;Porphyromonadaceae;Porphyromonas
290	Bacteria;Bacteroidetes;Bacteroidia;Bacteroidales;Porphyromonadaceae;Porphyromonas
291	Bacteria;Actinobacteria;Actinobacteria
292	Bacteria;Firmicutes;Clostridia;Clostridiales;Incertae Sedis XVIII;Symbiobacterium
293	Bacteria;Bacteroidetes;Flavobacteria;Flavobacteriales;Flavobacteriaceae
294	Bacteria;Firmicutes;Bacilli;Lactobacillales;Carnobacteriaceae;Granulicatella
295	Bacteria;Bacteroidetes;Bacteroidia;Bacteroidales;Prevotellaceae
296	Bacteria;Firmicutes;Clostridia;Clostridiales;Lachnospiraceae;Catonella
297	Bacteria;Firmicutes;Clostridia;Clostridiales;Veillonellaceae;Centipeda
298	Bacteria;Fusobacteria;Fusobacteria;Fusobacteriales;Fusobacteriaceae;Fusobacterium
299	Bacteria;Firmicutes;Clostridia;Clostridiales;Veillonellaceae;Veillonella
300	Bacteria;Proteobacteria
301	Bacteria;Actinobacteria;Actinobacteria;Actinomycetales;Corynebacteriaceae;Corynebacterium
302	Bacteria;Firmicutes;Bacilli;Lactobacillales;Streptococcaceae;Streptococcus
303	Bacteria;Firmicutes;Bacilli;Bacillales;Staphylococcaceae;Gemella
304	Bacteria;Firmicutes;Clostridia;Clostridiales;Veillonellaceae;Veillonella
305	Bacteria;Actinobacteria;Actinobacteria;Actinomycetales
306	Bacteria;Firmicutes;Clostridia;Clostridiales;Lachnospiraceae;Catonella
307	Bacteria;Spirochaetes;Spirochaetes;Spirochaetales;Spirochaetaceae;Treponema
308	Bacteria;Proteobacteria;Gammaproteobacteria;Legionellales;Legionellaceae;Legionella
309	Bacteria;Firmicutes;Clostridia;Clostridiales;Lachnospiraceae;Oribacterium
310	Bacteria
311	Bacteria
312	Bacteria;Firmicutes;Clostridia;Clostridiales;Eubacteriaceae;Eubacterium
313	Bacteria;Proteobacteria;Gammaproteobacteria;Chromatiales
314	Bacteria;Firmicutes;Bacilli;Bacillales;Bacillaceae;Bacillus
315	Bacteria;Firmicutes;Clostridia;Clostridiales;Lachnospiraceae;Oribacterium
316	Bacteria;Bacteroidetes;Flavobacteria;Flavobacteriales;Flavobacteriaceae;Capnocytophaga
317	Bacteria;Actinobacteria;Actinobacteria;Actinomycetales;Actinomycetaceae;Actinomyces
318	Bacteria
319	Bacteria;Acidobacteria;Acidobacteria_Gp6;Gp6
320	Bacteria
321	Bacteria
322	Bacteria;Firmicutes
323	Bacteria
324	Bacteria;Bacteroidetes;Bacteroidia;Bacteroidales;Prevotellaceae;Prevotella
325	Bacteria;Proteobacteria;Alphaproteobacteria;Sphingomonadales;Erythrobacteraceae;Porphyrobacter
326	Bacteria;Firmicutes;Clostridia;Clostridiales
327	Bacteria;Firmicutes
328	Bacteria;Firmicutes;Bacilli;Bacillales;Bacillaceae
329	Bacteria;Actinobacteria;Actinobacteria;Actinomycetales;Micrococcaceae
330	Bacteria;Bacteroidetes;Bacteroidia;Bacteroidales;Prevotellaceae;Prevotella
331	Bacteria
332	Bacteria;Firmicutes;Clostridia;Clostridiales;Veillonellaceae;Veillonella
333	Bacteria;Fusobacteria;Fusobacteria;Fusobacteriales;Leptotrichiaceae;Leptotrichia
334	Bacteria;Proteobacteria;Gammaproteobacteria;Pseudomonadales;Pseudomonadaceae;Pseudomonas
335	Bacteria;Actinobacteria;Actinobacteria;Actinomycetales;Corynebacteriaceae;Corynebacterium
336	Bacteria;Actinobacteria;Actinobacteria;Actinomycetales;Micrococcaceae;Rothia
337	Bacteria;Bacteroidetes;Sphingobacteria;Sphingobacteriales;Cytophagaceae;Hymenobacter
338	Bacteria;Firmicutes;Bacilli;Lactobacillales;Streptococcaceae;Streptococcus
339	Bacteria;Bacteroidetes;Bacteroidia;Bacteroidales;Prevotellaceae;Prevotella
340	Bacteria;Firmicutes;Bacilli;Lactobacillales;Carnobacteriaceae
341	Bacteria;Firmicutes;Clostridia;Clostridiales;Veillonellaceae
342	Bacteria;Bacteroidetes;Sphingobacteria;Sphingobacteriales;Sphingobacteriaceae;Nubsella
343	Bacteria;Proteobacteria;Gammaproteobacteria;Enterobacteriales;Enterobacteriaceae
344	Bacteria;Firmicutes;Bacilli;Bacillales
345	Bacteria;Proteobacteria;Alphaproteobacteria;Rhodobacterales;Rhodobacteraceae;Amaricoccus
346	Bacteria;Bacteroidetes;Bacteroidia;Bacteroidales;Porphyromonadaceae;Porphyromonas
347	Bacteria;Actinobacteria;Actinobacteria;Actinomycetales;Pseudonocardiaceae;Saccharopolyspora
348	Bacteria;Tenericutes;Mollicutes;Mycoplasmatales;Mycoplasmataceae;Mycoplasma
349	Bacteria;Proteobacteria;Alphaproteobacteria;Rhizobiales;Brucellaceae
350	Bacteria;Actinobacteria;Actinobacteria;Actinomycetales;Nocardioidaceae;Marmoricola
351	Bacteria;Bacteroidetes;Flavobacteria;Flavobacteriales;Flavobacteriaceae
352	Bacteria;Bacteroidetes;Bacteroidia;Bacteroidales;Porphyromonadaceae;Porphyromonas
353	Bacteria;Bacteroidetes;Sphingobacteria;Sphingobacteriales
354	Bacteria;Firmicutes;Clostridia;Clostridiales;Veillonellaceae;Veillonella
355	Bacteria
356	Bacteria;Firmicutes;Clostridia;Clostridiales;Eubacteriaceae;Eubacterium
357	Bacteria;Firmicutes;Clostridia;Clostridiales;Lachnospiraceae
358	Bacteria;Firmicutes;Clostridia;Clostridiales;Veillonellaceae;Selenomonas
359	Bacteria;Fusobacteria;Fusobacteria;Fusobacteriales;Leptotrichiaceae
360	Bacteria;Fusobacteria;Fusobacteria;Fusobacteriales;Fusobacteriaceae;Fusobacterium
361	Bacteria;Bacteroidetes;Bacteroidia;Bacteroidales;Prevotellaceae
362	Bacteria;Fusobacteria;Fusobacteria;Fusobacteriales;Leptotrichiaceae;Leptotrichia
363	Bacteria;Proteobacteria;Alphaproteobacteria;Rhodospirillales;Acetobacteraceae;Acetobacter
364	Bacteria;Firmicutes;Clostridia;Clostridiales;Peptostreptococcaceae;Peptostreptococcus
365	Bacteria;Proteobacteria;Gammaproteobacteria
366	Bacteria;Proteobacteria;Alphaproteobacteria;Sphingomonadales;Sphingomonadaceae;Sphingomonas
367	Bacteria;Firmicutes;Clostridia;Clostridiales
368	Bacteria;Proteobacteria;Alphaproteobacteria;Rhizobiales;Xanthobacteraceae
369	Bacteria;Actinobacteria;Actinobacteria;Actinomycetales;Microbacteriaceae;Microbacterium
370	Bacteria;Proteobacteria;Alphaproteobacteria;Rhodobacterales;Rhodobacteraceae
371	Bacteria
372	Bacteria
373	Bacteria;Bacteroidetes;Flavobacteria;Flavobacteriales;Flavobacteriaceae;Capnocytophaga
374	Bacteria;Firmicutes;Bacilli;Lactobacillales;Lactobacillaceae;Lactobacillus
375	Bacteria
376	Bacteria
377	Bacteria;Acidobacteria;Acidobacteria_Gp6;Gp6
378	Bacteria;Proteobacteria;Alphaproteobacteria
379	Bacteria;Bacteroidetes;Bacteroidia;Bacteroidales;Prevotellaceae;Prevotella
380	Bacteria;Bacteroidetes;Bacteroidia;Bacteroidales;Prevotellaceae;Prevotella
381	Bacteria;Firmicutes;Clostridia;Clostridiales;Peptostreptococcaceae;Peptostreptococcus
382	Bacteria;Firmicutes;Bacilli;Lactobacillales;Streptococcaceae;Streptococcus
383	Bacteria;Firmicutes;Erysipelotrichi;Erysipelotrichales;Erysipelotrichaceae;Catenibacterium
384	Bacteria;Firmicutes;Clostridia;Clostridiales;Veillonellaceae;Veillonella
385	Bacteria;Bacteroidetes;Bacteroidia;Bacteroidales;Prevotellaceae;Prevotella
