# Supplementary material for: Assessing Bacterial Populations in the Lung by Replicate Analysis of Samples from the Upper and Lower Respiratory Tracts
Source: PLoS One. 2012 Sep 6;7(9):e42786. doi: 10.1371/journal.pone.0042786 (PMC3435383; doi:10.1371/journal.pone.0042786)
Supplement: Table S3 — Values of θ and standard error for each sample estimated from the beta-binomial distribution model. (PDF) [file pone.0042786.s008.pdf]

Table S3

Subject ID	Location	Repeat	theta	se
Tx 20	BAL	Extraction	0.0107	0.0088
Tx 20	OralWash	Extraction	0.0039	0.0035
Tx 26	BAL	Extraction	0.007	0.0059
Tx 26	OralWash	Extraction	0.0054	0.0046
Tx 43	BAL	Extraction	0.0029	0.0025
Tx 43	OralWash	Extraction	0.0014	0.0013
Pulm 1	BAL	Extraction	0.0325	0.0316
Pulm 1	OralWash	Extraction	2.00E-04	2.00E-04
Pulm 3	BAL	Extraction	0.0028	0.003
Pulm 3	OralWash	Extraction	0.0014	0.0015
Pulm 4	BAL	Extraction	9.00E-04	0.001
Pulm 4	OralWash	Extraction	3.00E-04	4.00E-04
