# Supplementary material for: Assessing Bacterial Populations in the Lung by Replicate Analysis of Samples from the Upper and Lower Respiratory Tracts
Source: PLoS One. 2012 Sep 6;7(9):e42786. doi: 10.1371/journal.pone.0042786 (PMC3435383; doi:10.1371/journal.pone.0042786)
Supplement: Report S1 — Comparison of the single-sided outlier to other methods of community analysis. (PDF) [file pone.0042786.s009.pdf]

## Supplementary Report 1.

### Comparison of the single sided outlier method to other methods of community analysis

Below we contrast the single-sided outlier method reported here with several other reported methods to highlight the uses of each.

<u>Single-sided outlier method</u>

The single sided outlier method is designed to identify OTUs significantly enriched in one sample, which is lung in the examples studied here. The method can be used with replicates of each sample, resulting in a gain in power, but can also be used with as little as one replicate for each of the pair. The reason for focusing on this sampling approach is that in practice lung researchers are often confronted with BAL samples containing heavy admixture of upper airway bacteria, and often only a single oral wash sample is available for comparison. The Dirichlet-multinomial distribution is a natural choice for replicate samples because it separates the estimation of OTU proportions (Dirichlet) from the process of sampling (multinomial).

The multinomial distribution,

$$\text{Multinomial}(\vec{x} \mid \vec{p}) = \binom{N}{\vec{x}} \prod_i p_i^{x_i} ,$$

describes the probability of measuring a discrete set of OTU counts, $\vec{x}$, given the underlying OTU proportions, $\vec{p}$. The quantity inside the multinomial coefficient, *N*, is the total number of counts. If *n* is the number of OTUs, the multinomial distribution has *n-1* parameters, because the OTU proportions must sum to 1.

The input proportions to the multinomial distribution are unknown, and may vary to a degree between replicates. The Dirichlet distribution describes the likelihood of various proportions after a number of initial observations. It is parameterized by a vector of positive real numbers, $\vec{\alpha}$, having one element for each OTU:

$$\text{Dirichlet}(\vec{p} \mid \vec{\alpha}) = B(\vec{\alpha}) \prod_i p_i^{\alpha_i - 1} .$$

The normalizing factor, *B*, is the multivariate version of the beta function. The Dirichlet distribution has one more parameter than the multinomial distribution because the magnitude of the input vector is not constrained. The magnitude of $\vec{\alpha}$ provides a measure of variance for the resultant OTU proportions.

The two distributions may be combined to model a process of sampling from uncertain proportions. The Dirichlet-multinomial compound distribution takes all possible OTU proportions into account, weighting each by its likelihood under the Dirichlet distribution. Its analytical form is

$$\text{DirichletMultinomial}(\vec{x} \mid \vec{\alpha}) = \frac{\Gamma(A)}{\Gamma(N+A)} \prod_i \frac{\Gamma(x_i + \alpha_i)}{\Gamma(\alpha_i)},$$

where $A$ is the magnitude of the input vector and $\Gamma$ is the gamma function. For convenience, we use a separate quantity, $\theta = 1/(A+1)$, when discussing the extra-multinomial variance contributed by the uncertain distribution of OTU proportions. As this quantity approaches zero, the Dirichlet-multinomial distribution approaches the multinomial distribution.

To consider each OTU separately, we use the marginal form of the Dirichlet-Multinomial distribution, which is equivalent to a beta-binomial distribution with parameters $a = \alpha_i$ and $b = A - \alpha_i$ (see main text for equation). The marginal distribution for a single OTU describes the probability of observing a number of counts, considering all possible proportions for the other OTUs under the Dirichlet distribution. It constitutes the expected distribution for the null hypothesis that the counts of a single OTU arise from a Dirichlet-multinomial distribution estimated from the aggregate counts.

The Dirichlet-multinomial distribution has a simple interpretation, may be reduced to a convenient marginal form, and characterizes overdispersion with a single parameter. The finding that the Dirichlet-multinomial distribution fits the data reasonably adds power to the method of pair-wise comparisons.

Comparison to LEfSe

LEfSe is a valuable method that is particularly useful for identifying differential enrichment of taxa in settings where large numbers of samples are available for each of several conditions. However, in much of the work described here, the goal is to identify probable outliers in a small number of samples, and this is not the strength of LEfSe. Our method offers the following advantages for the problems described in this paper:

1. We are able to perform pairwise comparisons.
2. We test directly against the null hypothesis that OTU proportions are the same in both sample groups.
3. Outlier p-values are interpretable across sample sets. In LEfSe, taxa are filtered before the linear discriminant analysis (LDA) step, changing the magnitude of the effect size. The number of replicates also has an effect on LDA scores.

One important innovation of the LEfSe program is to aggressively filter OTUs before evaluating the effect size. We take the opposite approach for detecting outliers in paired samples, and use the complete set of OTUs as a yardstick for measuring outliers.

Comparison to RandomForrest

Machine learning methods have many advantages in identifying bacterial lineages enriched in one sample set compared to another. However, large

sample sizes are needed to use this approach effectively, and it is not suitable for use with OW/BAL pairs.

Dirichlet-multinomial mixtures

The Dirichlet-multinomial mixture (DMM) model from Holmes and Quince (PLoS One. 2012;7(2):e30126.) is another useful addition, but again focused mainly on a different purpose than identifying outliers in paired samples. The method is designed to distinguish underlying OTU proportions in multiple samples and identify the optimal number of components. This is valuable in the right context but not the job addressed by the single-sided outlier method reported here. Our null hypothesis is that the observed counts arise from a Dirichlet-multinomial model with only one mixture component.
